# Assessing UV Filter Exposure in Queensland, Australia: Human Biomonitoring Evidence Highlighting Homosalate in Children

**DOI:** 10.1021/acs.est.6c09445

**Published:** 2026-09-04

**Authors:** Katharina E. Ebert, Danielle E. Que, Anne Lotz, Thomas Brüning, Leisa-Maree L. Toms, Peter Hobson, Heath Glover, Holger M. Koch, Jochen F. Mueller, Daniel Bury

**Affiliations:** † Institute for Prevention and Occupational Medicine of the German Social Accident Insurance, Institute of the Ruhr University Bochum (IPA), Bürkle-de-la-Camp-Platz 1, Bochum 44789, Germany; ‡ Queensland Alliance for Environmental Health Sciences (QAEHS), 1974 The University of Queensland, 20 Cornwall Street, Woolloongabba, Queensland 4102, Australia; § School of Public Health and Social Work, Faculty of Health, 1969 Queensland University of Technology, Kelvin Grove, Queensland 4059, Australia; ∥ 10135 Sullivan Nicolaides Pathology, Bowen Hills, Queensland 4006, Australia; ⊥ Minderoo Centre–Plastics and Human Health, 20 Cornwall Street, Woolloongabba, Queensland 4102, Australia

**Keywords:** exposure assessment, risk assessment, human biomonitoring, UV filter, homosalate, 2-ethylhexyl salicylate, octocrylene

## Abstract

Sunscreens are an important protection measure against harmful effects of ultraviolet radiation. However, potential health effects of several commonly used UV filters are currently being discussed. We conducted a human biomonitoring (HBM)-based exposure and risk assessment for octocrylene (OC), 2-ethylhexyl salicylate (EHS), and homosalate (HMS; a mixture of *cis*- and *trans*-HMS) in urine samples collected in Queensland and across Australia from 2012 to 2023, pooled by age, sex, and collection period (187 pools representing approximately 18,700 individuals). The pooled urines were analyzed by LC–MS/MS for specific exposure biomarkers of these UV filters. Determinants of exposure were investigated using Tobit regression. Biomarker concentrations increased over time, and clear age trends were observed, with children showing roughly one order of magnitude higher concentrations than adolescents/adults. Concentrations of the EHS biomarker 5cx-EPS exceeded the health-based guidance value (HBM-I) in 23% of children’s pooled urines. For the *cis*-HMS biomarker HMS-CA5, most pooled urines of children exceeded HBM-I (80%) and 53% even HBM-II. These findings indicate the need to reduce HMS exposure in at least some individuals, confirming the necessity for stricter regulations for homosalate in Australia, as recently enforced in the European Union and recommended by Australian authorities, to ensure sunscreen safety.

## Graphical abstract

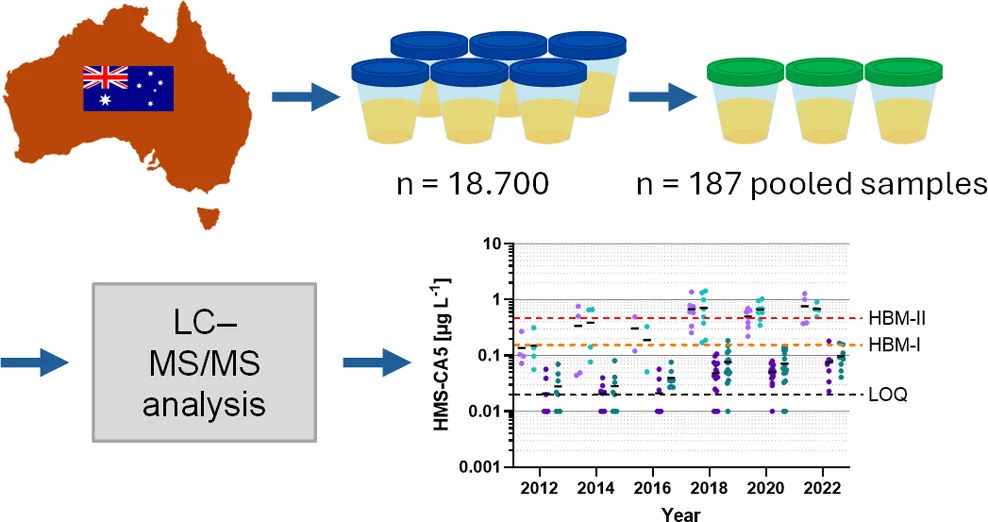

## 1 Introduction

Australia has one of the highest skin cancer incidence rates in the world.^1,2^ Ultraviolet (UV) radiation is widely considered the primary risk factor for skin cancer. Accordingly, there have been extensive endeavors to sensitize the Australian population to the dangers of UV radiation and to encourage appropriate sun protection measures.^3,4^ One of the central measures is the regular application of sunscreen, which enables the protection of areas not easily covered by clothing. The Australian Cancer Council advocates for the application of broad-spectrum, SPF50+, water-resistant sunscreen on days when the UV index reaches 3 and above, including reapplication every 2 h.^5^ However, in recent years, concerns have been raised regarding the human and environmental toxicity of several UV filters.^6–8^

Octocrylene (OC; octocrilene; 2-ethylhexyl 2-cyano-3,3-diphenyl acrylate; CAS registry no. 6197-30-4), 2-ethylhexyl salicylate (EHS; octyl salicylate; octisalate; CAS registry no. 118-60-5), and homosalate (HMS, homomenthyl salicylate; CAS registry no. 118-56-9) are UV filters commonly used worldwide in sunscreens and personal care products.^9–11^ Maximum permitted concentrations of OC and EHS are 10% and 5% respectively in Australia, the U.S.A., the Association of Southeast Asian Nations (ASEAN), and the European Union. HMS is currently permitted up to 15% in the U.S.A and Australia, however, the European Union and ASEAN have recently lowered the maximum concentration from 10% to 7.34% and restricted its usage to face products (with the exception of propellant spray products) due to concerns about nephrotoxicity.^12–16^ The Australian Industrial Chemicals Introduction Scheme and the Australian Therapeutic Goods Administration similarly recommended stricter regulations for homosalate in an evaluation statement on homosalate in December 2024 and in a safety review of seven UV filters in July 2025, respectively, both citing insufficient margins of safety.^17,18^

The systemic absorption of OC, EHS, and HMS after sunscreen application has been proven in multiple studies.^19–25^ Exposure occurs predominantly via the dermal pathway, but additional oral exposure (e.g., via lip care products, hand-to-mouth contact or eating food with bare hands after applying sunscreen) is also likely, especially for children. As for other compounds with dermal and mixed-route exposures, human biomonitoring (HBM) is the ideal choice for robust exposure assessments.^26^ The determination of metabolites (and not the parent compounds) as exposure biomarkers is preferable when analyzing personal care product ingredients in order to avoid the risk of (pre)­analytical contamination,^27^ and urine is the preferred matrix for the analysis of short-lived xenobiotics such as OC, EHS, and HMS, which are rather rapidly metabolized and excreted.^19,20,25^ We previously described specific metabolites of the above-mentioned UV filters in urine as appropriate exposure biomarkers; CPAA and DOCCA for OC and 5cx-EPS and 5OH-EHS for EHS.^20,28^ In the case of HMS, which is produced and used as an isomeric mixture,^29^ we identified HMS-CA5 and 3OH-cHMS as specific biomarkers for *cis*-HMS (cHMS) and tHMS-CA and 3OH-tHMS for *trans*-HMS (tHMS) (see the materials and methods section for the full chemical names and Figure S1 for the chemical structures). We developed analytical methods for their determination in urine^19,30–32^ and investigated their toxicokinetics after both oral and dermal exposure.^19,20,25,28,29^ Urinary excretion fractions were derived from the metabolism data which allow the back-calculation of urinary metabolite biomarker concentrations to external doses in the form of oral-dose equivalent intakes.

For the key biomarkers of these UV-filters, the German Human Biomonitoring Commission has derived health-based HBM guidance values (German HBM-I and -II values^33,34^ ), which allow a direct risk assessment based on measured urinary biomarker concentrations.^35–38^ These HBM values have previously been applied in population studies in 24 h urine samples from the German Environmental Specimen Bank (ESB).^39^

In the present study, we analyzed pooled urine samples from an Australian population (with an oversampling of Queensland) that have previously been analyzed e.g. for biomarkers of plasticizers, bisphenol A, benzotriazole and benzothiazole UV stabilizers, and organophosphate esters.^40–44^ These samples are surplus pathology samples collected as part of routine clinical tests. Although they are not statistically representative of the Australian population, according to previous studies, there are no indications that concentrations of exposure biomarkers in surplus pathology samples differ considerably from those in the general population.^40,45^ In total, the studied samples represent individual spot urines from approximately 18,700 Australians and had been pooled by six age groups, by sex, and by sample collection period (2 year periods, beginning with 2012/13 and ending with 2022/23), with each pooled urine consisting of 100 individual spot urines. While pooled samples do not support exposure and risk assessment at the individual level and only convey limited information about population variance and percentiles, they provide an estimate of central tendencies in a population and may also highlight time trends or differences within the population (e.g., by sex or age) (see Heffernan et al.^45^ for an in-depth discussion). Thereby their use represents a powerful and cost-efficient approach for detecting population-relevant exposures, identifying key risk drivers, and informing more detailed, focused population-level HBM programs.

## 2 Materials and Methods

### 2.1 Study Design

Deidentified individual human urine samples were obtained from surplus pathology samples collected by the pathology laboratory Sullivan Nicolaides Pathology (SNP, Bowen Hills, Queensland, Australia) between 2012 and 2023 in 2 year periods (2012–2013, 2014–2015, ...) from individuals of the Australian population as part of routine clinical tests which are community-based pathology tests ordered by an individual’s healthcare provider as part of standard patient care. Only a low percentage (<8%) of pathology urine samples received at SNP comes from hospitals. The majority of these samples are submitted for suspected urinary tract infection (∼50%), diabetes and metal screening/monitoring, most of which show no abnormal pathology or are within the normal ranges. Due to sample availability, the study population was skewed toward Queensland (92% of the samples), with most samples (75%) originating from Southeast Queensland (including Brisbane, Gold Coast, Sunshine Coast, Toowoomba, etc., which cover about 75% of the Queensland population), possibly affecting the representativeness of the Australian population. Although there is a predominance of samples from Southeast Queensland, the rest of samples came from all over Australia.

Surplus individual spot urine samples were pooled by the Queensland Alliance for Environmental Health Sciences (QAEHS) and Queensland University of Technology (QUT), with only sex, date of birth, and sample collection date data retained, maintaining patient anonymity. This available information was used to ensure that no individual contributed more than one sample to the pools in each sampling round. However, we cannot exclude the possibility that some individuals contributed samples across multiple collection rounds, although the likelihood of this occurring is low.

As all samples were deidentified and pooled in the pathology laboratory before analysis, any per-sonal and identifiable information is removed, therefore a consent protocol is not required. The ap-proval for the ethics of this study was granted by The University of Queensland Medical Research Ethics Committee (2013000317). As per the ethics committee guidelines, all samples and obtained analytical results were stored in a secured facility and privately held database.

Samples were stratified based on sex (male and female), age (0–5, 6–15, 16–30, 31–45, 46–60, and ≥61 years old), and sample collection period (six 2 year periods from 2012 to 2023). Individual urine samples were pooled based on equal volume, with each pool corresponding to 100 individuals. A total of 18,700 individual samples were pooled into 187 pools.

### 2.2 Chemical Analysis

The metabolites of OC (2-cyano-3,3-diphenylacrylic acid (CPAA) and 2-(carboxymethyl)­butyl 2-cyano-3,3-diphenyl acrylate (dinor OC carboxylic acid; DOCCA)), EHS (5-(((2-hydroxybenzoyl)­oxy) methyl)­heptanoic acid (5cx-EPS) and 2- ethyl-5-hydroxyhexyl 2-hydroxybenzoate (5OH-EHS)), *trans*-HMS ((1*S*,5*R*)/(1*R*,5*S*)-5-((2-hydroxybenzoyl)­oxy)-3,3-dimethylcyclohexane-1-carboxylic acid (tHMS-CA) and (1*R*,3*S*)/(1*S*,3*R*)-3-hydroxy-3,5,5-trimethylcyclohexyl 2-hydroxybenzoate (3OH-tHMS)) and *cis*-HMS ((1R,3S,5S)/(1S,3R,5R)-3-((2-hydroxybenzoyl)­oxy)-1,5-dimethylcyclohexane-1-carboxylic acid (HMS-CA5) and (1*R*,3*R*)/(1*S*,3*S*)-3-hydroxy-3,5,5-trimethylcyclohexyl 2-hydroxybenzoate (3OH-cHMS)) were quantified using two online-SPE-LC-MS/MS methods and stable isotope dilution analysis. Previous methods for these metabolites have already been published^32,39^ but had to be optimized due to recent changes in product quality affecting the OC method (see the Supporting Information Section S2 for more details). We developed a new analytical method for the OC biomarkers with improved retention time stability, and improved chromatographic separation of CPAA from matrix components. The EHS biomarkers, previously measured together with the OC metabolites, were instead included in the HMS method. See Section S2.1 of the Supporting Information for the used chemicals and reagents. Calibration was performed in water. Sample preparation was performed as described previously.^32,39^ In short, urine samples were thawed and homogenized after reaching room temperature by inverting several times. Three hundred microliter aliquots were transferred into silanized 1.5 mL HPLC vials. Thirty microliter of internal standard solution (final concentrations in the sample: 20 μg L^–1^ CPAA-*d*_10_, 2 μg L^–1^ 5OH-EHS-*d*_4_, 1 μg L^–1^ DOCCA-*d*_10_ and 5cx-EPS-*d*_4_, 0.5 μg L^–1^ tHMS-CA-*d*_4_ and cHMS-CA-*d*_4_), 100 μL of 1 M ammonium acetate solution (pH 6–6.4) and 6 μL of β-glucuronidase from *E. coli* K12 (premixed 1:1 (v/v) with ammonium acetate solution, ∼0.4 u) were added. Samples were thoroughly mixed by inverting and incubated in a water bath at 37 °C for 3 h. Thirty microliter of formic acid were added and the samples were mixed, frozen overnight at −20 °C, thawed, centrifuged at 1900*g* for 10 min and the supernatant was transferred into new silanized HPLC vials and used for analysis. Calibration samples and quality control (QC) samples (see the Supporting Information, Section S2.1) were treated identically to urine samples.

Sample analysis was performed using an Agilent 1260 Infinity II HPLC system (Agilent Technologies, Waldbronn, Germany) coupled to a 5500 Triple Quad mass spectrometer (Sciex, Darmstadt, Germany). The sample order was randomized.

For continuous quality control, three pooled urine samples with known biomarker concentrations were analyzed twice in each analytical batch, once after the calibration curve and once at the end of the batch of at maximum 50 samples. These quality control samples had been prepared from naturally biomarker-containing urine samples, in part spiked to obtain target biomarker concentrations. The validity of each analytical batch was evaluated according to the guidelines of the working group “analyses in biological materials” of the Permanent Senate Commission for the Investigation of Health Hazards of Chemical Compounds in the Work Area of the German Research Foundation (Deutsche Forschungsgemeinschaft).^46^ As an additional quality control criterion, quantifier/qualifier ion ratios were calculated for each peak and compared to the arithmetic mean of the calibration samples. Deviations exceeding the maximum permitted tolerances listed in decision 2002/657/EC^47^ required reanalysis of the sample. Unusually strong quenching of the CPAA internal standard, accompanied by fronting chromatographic peaks, were observed in several samples without the ion ratios being affected. These samples were reanalyzed in dilution using a fresh sample preparation. The diluted samples showed normal CPAA-*d*_10_ peak shapes and intensities; hence, CPAA concentrations were calculated from these diluted samples. Samples with biomarker concentrations outside of the calibration range were similarly reanalyzed in dilution.

LOQs in urine were 0.5 μg L^–1^ for CPAA, 0.1 μg L^–1^ for DOCCA and 5OH-EHS, 0.04 μg L^–1^ for 3OH-cHMS, 0.03 μg L^–1^ for tHMS-CA, and 0.02 μg L^–1^ for 5cx-EPS, HMS-CA5, and 3OH-tHMS. Intra- and interday imprecision were <8% for all metabolite biomarkers. Mean relative recoveries were between 84% and 110% for all analytes. See Section S2 of the Supporting Information for detailed descriptions of the instrument setup, the analytical methods, validation data, and quality control.

### 2.3 Data and Statistical Analysis

SciexOS 3.4.0. (Sciex, Darmstadt, Germany) was used for instrument control and quantitative data analysis of LC–MS/MS measurements. Correlations between metabolite concentrations were calculated by Spearman’s rank using IBM SPSS Statistics Version 28.0.1.1. Tobit regression was performed to investigate predictors of UV filter exposure using R version 4.6.1 and the R packages haven, tidyverse, sjlabelled, rstatix, and AER. Urinary biomarker concentrations in μg L^–1^ were used for statistical analysis. Although adjustment to urinary creatinine or specific gravity are common in HBM to account for urinary dilution, these might introduce bias when comparing between age groups and sexes: specific gravity and urinary creatinine are dependent on sex, and urinary creatinine is dependent on age.^48,49^ Furthermore, in pooled urines, the variability in urinary dilution is rather low.^44^ Therefore, creatinine- or specific gravity-adjustment was not performed. Natural log­(ln)-transformed concentrations were used for regression models to approximate a normal distribution. Potential predictors were age (categorical), sex, and sample collection period (categorical; referred to as “year” in the tables). Year was alternatively treated as continuous variable in the forms of *x* (linear), *x* ^1/2^, ln­(*x*), *x* ^2^, *x* ^3^, *x* ^–1^, and *x* ^–2^. An age*sex interaction term was included in the initial models. A likelihood-ratio-test was performed to evaluate whether the interaction term significantly improved the model; if not, the interaction term was removed from the final model. To facilitate the interpretation of models with an interaction term, we constructed linear contrasts to estimate the effect of sex (female vs male) within each age group (termed “age-specific sex effects”). These contrasts correspond to exp­(β_Sex_) × exp­(β_Age*Sex_). Biomarker concentrations below the LOQ were set to LOQ/2 for Spearman rank correlation and data visualization. For descriptive statistics, geometric means and medians of biomarker concentrations for strata with concentrations below the LOQ in at least one pooled urine were calculated using a best-case/worst-case assessment. For this purpose, concentrations below the LOQ were set to either 0 or the LOQ. If these two approaches resulted in different concentrations, medians and GMs were reported as being smaller than the value calculated using the worst-case approach (i.e., with the concentration set to the LOQ). Data visualization was performed using Graphpad Prism 10.5.0. Microsoft Excel for Microsoft 365 MSO (Microsoft Corporation, Redmond, WA, USA) was used for all other calculations.

## 3 Results and Discussion

### 3.1 Concentrations of UV Filter Biomarkers in Australian Pooled Urine Samples

Table 1 shows the descriptive statistics of the biomarker concentrations measured in the Australian pooled urine samples (see Table S9 in the Supporting Information for the separate descriptive statistics for a younger group (≤15 years old, henceforth referred to as children) and an older group (≥16 years old, encompassing adolescents and adults) for each sample collection period). See Figures 1 and 2 for a graphical presentation of the data, plotted either against the sample collection period or the different age groups. In 100% of the children’s pools, concentrations of the most sensitive biomarker of each investigated UV filter were above the LOQ. Also in adults, 100% of the samples had UV filter biomarker concentrations above the LOQ for the OC biomarker CPAA and the EHS biomarker 5cx-EPS, and also for the tHMS biomarker tHMS-CA, except in 2012–2013 (94% above the LOQ). Concentrations of the cHMS biomarker HMS-CA5 were above the LOQ in 100% of the samples from the most recent 2 year period. For comparison, median concentrations of CPAA and 5cx-EPS in the pooled urine samples exceeded the 95th percentiles of concentrations of these biomarkers in urine samples from the ESB, collected from German students in winter. These UV filter biomarker concentrations in the ESB samples are expected to constitute background exposures to OC and EHS, mainly resulting from sources other than sunscreen use.^39^ The correlation between these major biomarkers and their confirmatory biomarkers was excellent (Spearman’s rho of 0.891 for CPAA and DOCCA, 0.927 for 5cx-EPS and 5OH-EHS, 0.953 for tHMS-CA and 3OH-tHMS, and 0.847 for HMS-CA5 and 3OH-cHMS, with *p* < 0.001, respectively; Figures S5 and S6). For the sake of conciseness, the following trend analyses will therefore focus on the major biomarkers only. It should be noted that for comparison of exposures between UV filters, a direct comparison of biomarker concentrations is not sufficient, but instead, the respective urinary excretion fractions need to be accounted for (which for example results in roughly two orders of magnitude higher biomarker concentrations for CPAA compared to 5cx-EPS at the same external dose of the parent compounds).

**Table 1:** Descriptive Statistics of the Entire Samples Set (*n* = 187) for both Major and Confirmatory Biomarkers of the Investigated UV Filters

parent	metabolite	*n* > LOQ (%)	min [μg L^–1^]	median [μg L^–1^]	GM [μg L^–1^]	max [μg L^–1^]
OC	CPAA^a^	187 (100)	1.65	37.0	44.0	503
	DOCCA^b^	162 (87)	<LOQ [0.1]	0.267	<0.408	6.08
EHS	5cx-EPS^a^	187 (100)	0.093	1.07	1.19	14.5
	5OH-EHS^b^	186 (99)	<LOQ [0.1]	0.717	<0.696	5.63
tHMS	tHMS-CA^a^	186 (99)	<LOQ [0.03]	0.345	<0.352	2.40
	3OH-tHMS^b^	152 (81)	<LOQ [0.02]	0.041	<0.054	0.434
cHMS	HMS-CA5^a^	161 (86)	<LOQ [0.02]	0.060	<0.087	1.44
	3OH-cHMS^b^	70 (37)	<LOQ [0.04]	<LOQ [0.04]	<0.063	0.496

a Major (most sensitive) biomarker.

b Confirmatory biomarker.

**Figure 1:**
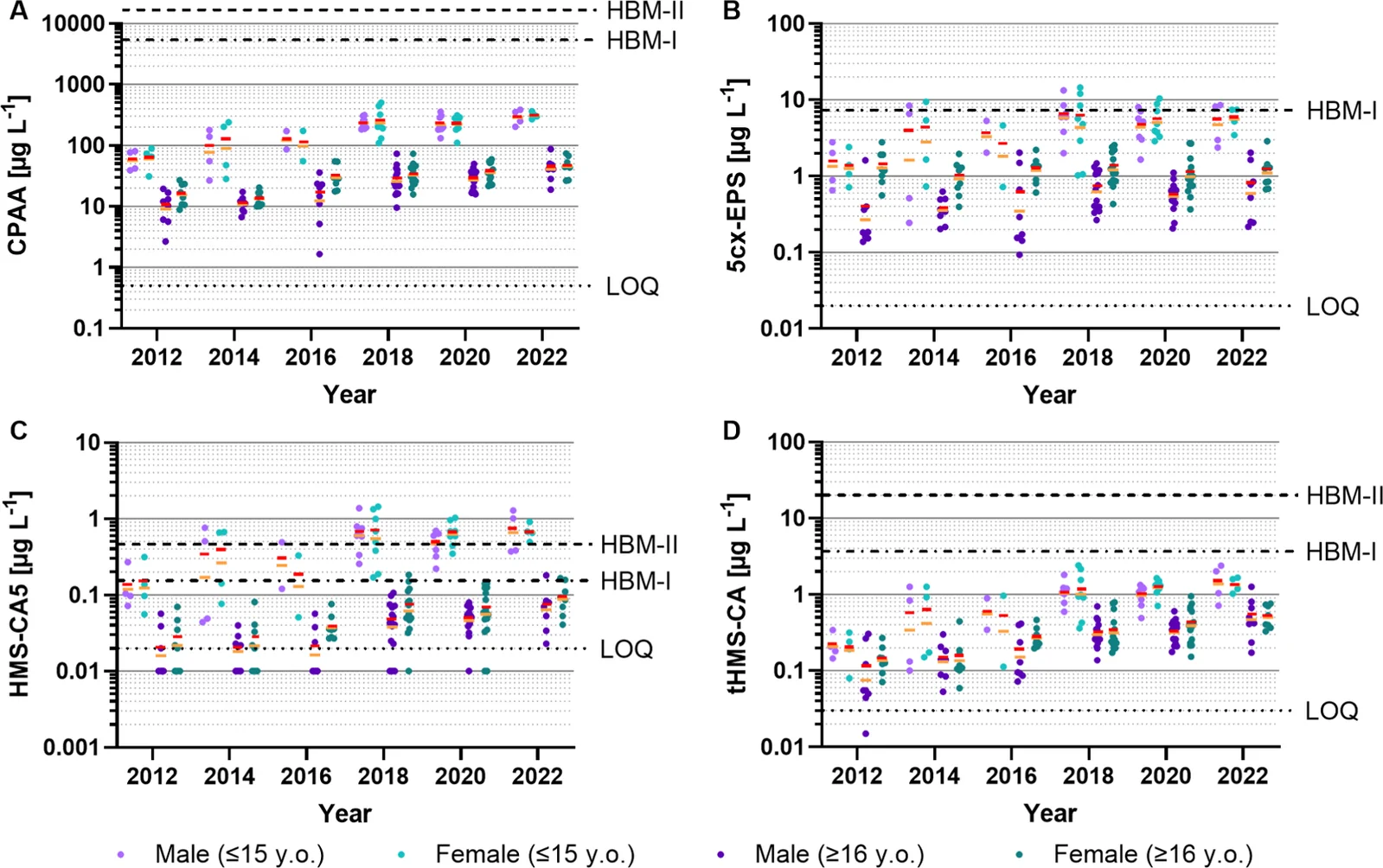
Concentrations of the main urinary biomarkers of (A) OC, (B) EHS, (C) *cis*-HMS and (D) *trans*-HMS in the investigated pooled urine samples plotted against the sample collection period. The labels of the *x*-axis show the beginning of each sample collection period (e.g., 2012 for the period 2012/13). The six age strata were combined to roughly differentiate between children (≤15 years old) and adolescents/adults (≥16 years old). Each data point represents a single pooled urine sample and orange and red lines show the geometric and arithmetic means, respectively. Black dotted lines show the LOQ of the respective biomarker, while black dotted dashed and dashed lines show the HBM-I and HBM-II derived by the German Human Biomonitoring Commission. For simplicity, only the HBM values for men/children are shown.

**Figure 2:**
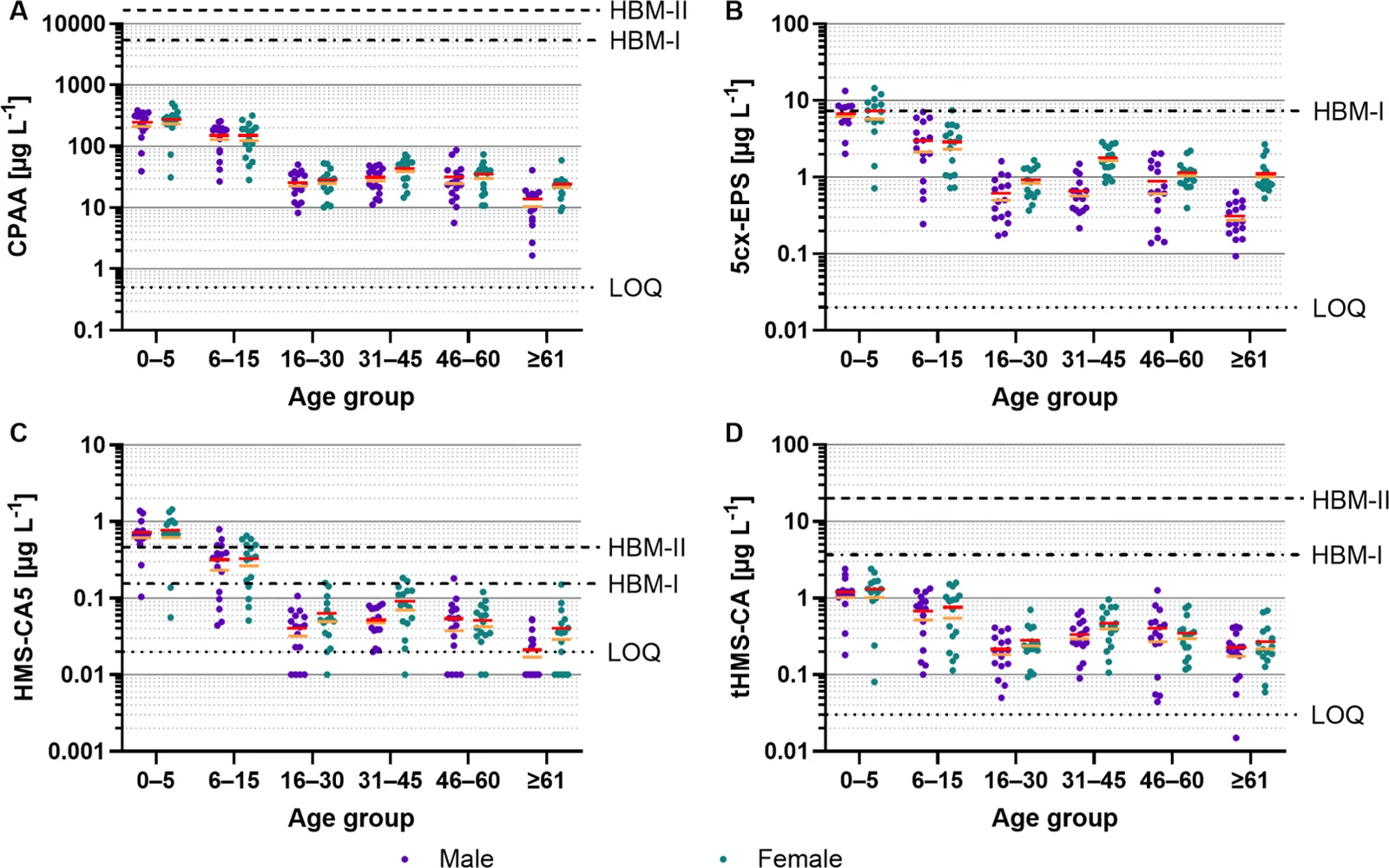
Concentrations of the main urinary biomarkers of (A) OC, (B) EHS, (C) *cis*-HMS and (D) *trans*-HMS in the investigated pooled urine samples plotted against the age strata. Each data point represents a single pooled urine sample and orange and red lines show the geometric and arithmetic means, respectively. Black dotted lines show the LOQ of the respective biomarker, while black dotted dashed and dashed lines show the HBM-I and HBM-II derived by the German Human Biomonitoring Commission. For simplicity, only the HBM values for men/children are shown.

### 3.2 Influence of Age, Sex and Sampling Period on UV Filter Exposures in Australian Pooled Urines

To identify and quantitatively investigate potentially intercorrelated determinants of UV filter exposure, a regression model was developed. Biomarker concentrations were ln-transformed to approximate a normal distribution. Age, sex, and sampling period were assumed to be likely determinants and were thus considered in the model. Male adults aged 61 years and older from the sample collection period 2012–2013 were used as reference (see Section S5 of the Supporting Information for the underlying rationale). It was postulated that sunscreen and cosmetics usage showed different age trends for the two sexes. As such, an age*sex interaction term was included in the initial model (Table S10). A likelihood-ratio-test showed that the interaction term significantly improved the model for CPAA and 5cx-EPS, but not for tHMS-CA and HMS-CA5 (Table S11). Hence, the interaction term was only included for CPAA and 5cx-EPS in the final model. For comparison between models treating year as categorical or continuous variable, the Akaike information criterion (AIC) was applied, as it includes a penalty for model complexity. Having the lowest AIC value, the categorical model was best suited for CPAA and HMS-CA5 based on this criterion. In contrast, for 5cx-EPS and tHMS-CA certain forms of treating year as continuous variable resulted in lower AIC values. Judging by Δ_*i*_ (i.e., the difference between the AIC value of the model in question and the lowest AIC value), the difference between models was low (5cx-EPS; Δ_*i*_ < 3 for the categorical model) to moderate (tHMS-CA; Δ_*i*_ = 4.0). Furthermore, concentrations of 5cx-EPS, HMS-CA5, and tHMS-CA in children did not follow a strictly monotonous time trend (Figure 1), thus contradicting treatment of year as continuous variable. Finally, the choice of model did not affect interpretations. Therefore, we treated all four biomarkers uniformly and modeled year as categorical. For the final models, the obtained effect sizes (exp­(β)), 95th confidence intervals, and p-values are shown in Table 2 (for comparison, this type of information is reported in Tables S12 and S13 for those models with lower AIC values for 5cx-EPS and tHMS-CA). Because the outcome variable was measured in pooled urine samples, the regression coefficients describe associations at the pool (group) level rather than at the individual level. Consequently, the observed relationships should be interpreted as differences in average biomarker concentrations between pools representing different demographic groups and sampling periods, and not as individual-level associations.

**Table 2:** Potential Determinants (Predictors) of Biomarker Concentrations in Australian Pooled Urine Samples, Determined Using Tobit Regression^b^

	CPAA				5cx-EPS				tHMS-CA			HMS-CA5		
factor	exp(β)	95% CI	*p* value		exp(β)	95% CI	*p* value		exp(β)	95% CI	*p* value	exp(β)	95% CI	*p* value
intercept	4.69	(3.74–5.87)			0.171	(0.120–0.244)			0.066	(0.0512–0.0851)		0.00907	(0.00660–0.0125)	
age														
≥0–<5	19.6	(15.0–25.4)	**<0.001**		22.3	(14.7–33.8)	**<0.001**		4.98	(3.90–6.35)	**<0.001**	23.9	(18.1–31.7)	**<0.001**
≥5–<15	12.3	(9.58–15.9)	**<0.001**		7.75	(5.20–11.5)	**<0.001**		2.68	(2.12–3.39)	**<0.001**	10.2	(7.76–13.3)	**<0.001**
≥15–<30	2.15	(1.67–2.77)	**<0.001**		1.82	(1.22–2.72)	**0.003**		1.06	(0.837–1.34)	0.629	1.72	(1.31–2.27)	**<0.001**
≥30–<45	2.61	(2.03–3.36)	**<0.001**		2.08	(1.40–3.10)	**<0.001**		1.72	(1.36–2.17)	**<0.001**	2.41	(1.84–3.15)	**<0.001**
≥45–<60	2.37	(1.84–3.05)	**<0.001**		2.22	(1.49–3.31)	**<0.001**		1.43	(1.13–1.80)	**0.003**	1.72	(1.31–2.27)	**<0.001**
≥61	1	ref	ref		1	ref	ref		1	ref	ref	1	ref	ref
sex = male	1	ref	ref		1	ref	ref		1	ref	ref	1	ref	ref
sex = female	2.05	(1.59–2.64)	**<0.001**		3.69	(2.48–5.49)	**<0.001**		1.17	(1.02–1.34)	**0.026**	1.31	(1.12–1.53)	**<0.001**
age*sex				age-specific sex effects^a^				age-specific sex effects^a^						
*F*; ≥0–<5	0.539	(0.372–0.780)	**0.001**	1.10	0.252	(0.141–0.452)	**<0.001**	0.930						
*F*; ≥5–<15	0.470	(0.329–0.672)	**<0.001**	0.964	0.295	(0.168–0.519)	**<0.001**	1.09						
*F*; ≥15–<30	0.546	(0.381–0.783)	**<0.001**	1.12	0.460	(0.260–0.811)	**0.007**	1.70						
*F*; ≥30–<45	0.693	(0.485–0.990)	**0.044**	1.42	0.777	(0.442–1.36)	0.379	2.87						
*F*; ≥45–<60	0.590	(0.413–0.843)	**0.004**	1.21	0.464	(0.264–0.816)	**0.008**	1.71						
*F*; ≥61	1	ref	ref	2..05	1	ref	ref	3.69						
year														
2012–2013	1	ref	ref		1	ref	ref		1	ref	ref	1	ref	ref
2014–2015	1.14	(0.924–1.40)	0.226		1.16	(0.839–1.61)	0.367		1.47	(1.12–1.92)	**0.006**	1.29	(0.942–1.78)	0.111
2016–2017	1.83	(1.47–2.27)	**<0.001**		1.50	(1.06–2.12)	**0.021**		2.23	(1.68–2.97)	**<0.001**	1.60	(1.14–2.24)	**0.006**
2018–2019	2.87	(2.40–3.43)	**<0.001**		2.03	(1.53–2.69)	**<0.001**		3.55	(2.81–4.49)	**<0.001**	3.18	(2.41–4.19)	**<0.001**
2020–2021	2.97	(2.48–3.55)	**<0.001**		1.78	(1.34–2.36)	**<0.001**		4.00	(3.16–5.05)	**<0.001**	3.20	(2.43–4.22)	**<0.001**
2022–2023	4.06	(3.30–4.99)	**<0.001**		1.96	(1.41–2.71)	**<0.001**		5.32	(4.06–6.97)	**<0.001**	4.31	(3.15–5.88)	**<0.001**

a exp­(β_Sex_) × exp­(β_Age*Sex_).

b The reference group is male, ≥61 years old, sample collection period 2012–2013. *p* values <0.05 are shown in bold.

The large percentage of pooled urine samples with biomarker concentrations above the LOQ suggests widespread exposure to UV filters in the studied Australian population. Clear age trends were observed for all biomarkers, with children showing significantly higher (factors 2.6–24) biomarker levels than the reference group (i.e., age ≥61) and, in comparison, only moderate differences between age groups in adults. This trend could be explained by the increased sun protection measures such as sunscreen use of children compared to adults. Children’s skin is considered particularly vulnerable to the damaging effects of UV radiation.^50^ Australian schools and early childhood education and care centers are encouraged to join the national SunSmart programs and to develop specific sun protection policies.^51^ For Queensland state schools, sun protection policies are even mandatory and they must provide their students with sun protection factor (SPF) ≥ 30 broad-spectrum water-resistant sunscreen to use.^52^ Furthermore, this trend could also indicate higher UV filter absorption in children because they have a proportionally larger body surface area to body mass ratio than adults and, at least up to 6 years of age, thinner skin and a lower barrier function than adults.^53,54^ It might also indicate additional uptakes of children due to mouthing objects and hand-to-mouth contact.^55^

For the HMS biomarkers tHMS-CA and HMS-CA5, sex differences were low to moderate (17% and 31%, respectively). For OC and EHS, the model was improved by the introduction of an age*sex interaction term, indicating age-dependency of sex differences (see column ‘age-specific sex effects’ for an estimate of the total effect considering both the predictor sex and the interaction term Age*Sex). In children, no major sex differences were observed (10% at maximum). In contrast, particularly strong sex differences were observed for 5cx-EPS in adults, with female adults’ pooled urines having, in tendency, higher 5cx-EPS concentrations (up to about a factor of 3.7). For CPAA, pronounced sex differences were observed in some age groups (up to a factor of 2.1). The identification of exposure sources is outside the scope of this study, but the fact that similarly strong sex differences were not observed in adults for tHMS-CA and HMS-CA5 may indicate an additional exposure source for EHS (and potentially also OC) that is not sunscreen usage, possibly other personal care products that are used more by women.

Concentrations of CPAA, tHMS-CA, and HMS-CA5 increased steadily from 2012–2013 to 2022–2023, as can be seen from the increasing effect sizes in Table 2. In the case of HMS-CA5, this also involved an increase in detection frequencies from 50% of pooled urine samples >LOQ in 2012 to 100% in 2022 (see Table S9). In the case of 5cx-EPS, concentrations also increased from 2012 to 2018 but remained relatively stable afterward.

It has been reported that the SPF of sunscreens applied by U.S. American adults gradually increased between 2005 and 2015.^56^ A similar development may have occurred in Australia, leading to higher UV filter concentrations in sunscreens and, in turn, to higher population exposure. Furthermore, increasing exposures to the investigated UV filters might also result from substitution of certain UV filters, such as benzophenones which have been regulated. Finally, although the SunSmart program was already established in the 1980s, compliance in the population may still be increasing; for example, increased compliance with sunscreen practices was reported in Australian early childhood services between 2008 and 2018.^57^

### 3.3 Implications for Risk Assessment and Risk Management

As mentioned above, the German Human Biomonitoring Commission has derived toxicology-based threshold values for all four of the herein investigated biomarkers, using human toxicokinetic data and the most sensitive end point from animal experiments. The HBM-I value represents the urinary biomarker concentration at and below which no health effects are expected according to the current state of knowledge, and no action is required. Conversely, the HBM-II value is the limit value at which and above which adverse health effects are possible and thus an acute need for exposure reduction and provision of biomedical advice is given.^34^ HBM values have been derived separately for women on one hand and men and children on the other, due to differences in body-weight-dependent median urine output between women and children/men (Table 3).^35–38,58^ Figure 1 includes HBM-I and HBM-II values for men and children as dotted lines for ease of comparison. On a side note, the HBM-I and -II values for OC do not consider potential contamination of sunscreens with benzophenone resulting from OC degradation,^59,60^ and this topic exceeds the scope of the present study.

**Table 3:** Health-Based HBM Guidance Values (HBM-I and HBM-II Values) derived by the German Human Biomonitoring Commission for the Major UV Filter Biomarkers Investigated in this Study and Percentages of Samples Exceeding these Guidance Values

UV filter	OC^36,37^	EHS^35,37^	cHMS^38^	tHMS^38^
critical effects	effects on reproduction and development	prolonged pregnancy, decreased gestational index, and increased postimplantation loss	adverse effects in the kidneys	
biomarker	CPAA	5cx-EPS	HMS-CA5	tHMS-CA
HBM-I				
women	4.5 mg L^–1^	6.4 μg L^–1^	0.14 μg L^–1^	3.2 μg L^–1^
men/children	5.3 mg L^–1^	7.4 μg L^–1^	0.16 μg L^–1^	3.7 μg L^–1^
HBM-II				
women	16 mg L^–1^	-	0.42 μg L^–1^	9.5 μg L^–1^
men/children	18 mg L^–1^	-	0.49 μg L^–1^	11 μg L^–1^
percentage of samples exceeding HBM-I				
children	0%	23%	80%	0%
adults	0%	0%	6%	0%
men	0%	0%	2%	0%
women	0%	0%	10%	0%
percentage of samples exceeding HBM-II				
children	0%	-	53%	0%
adults	0%	-	0%	0%
men	0%	-	0%	0%
women	0%	-	0%	0%

No exceedances of the respective HBM-I values were observed for CPAA in all age groups, or for 5cx-EPS in adults (Table 3). While maximum CPAA concentrations remained roughly a factor of 10 below the HBM-I value, maximum 5cx-EPS concentrations in adults corresponded to 42% of the HBM-I value. However, higher biomarker concentrations in individual urine samples, possibly exceeding these thresholds, cannot be excluded. Furthermore, considering that the HBM-I value for the EHS biomarker is based on reproductive and/or developmental toxicity, the in tendency higher exposures in women (Section 3.2) should not go unnoticed. In children’s pooled urines, the HBM-I value for 5cx-EPS was exceeded in several samples (23%), indicating that exposure levels were above the concentration at and below which no adverse health effects are expected.

Most strikingly, however, the majority (80%) of children’s pools (and a low number of adult pools; 6%; with women having a higher percentage of exceedances, which will at least in part be related to, in tendency, higher concentrations compared to men, see Section 3.2) exceeded the HBM-I value for the *cis*-HMS biomarker HMS-CA5. From 2018 on, all children’s pools consistently exceeded the HBM-I. Furthermore, about half of the children’s pools (53%) even exceeded the HBM-II value, with first exceedances going back as far as 2016. For the *trans*-HMS biomarker tHMS-CA, we observed no exceedances of the HBM guidance values in the pooled urine samples; however, individual contributors may still have had biomarker concentrations exceeding these values. This might seem surprising at first glance but is related to (i) differences in relative biological availabilities of *cis*- and *trans*-HMS for the dermal and oral pathways and (ii) the lack of toxicological data for diastereomerically pure *cis*- or *trans*-HMS (for a detailed discussion, see Section S6 in the Supporting Information). This lack of toxicological data for pure *cis*- and *trans*-HMS complicates risk assessment because it hampers differential risk assessment of tHMS and cHMS, irrespective of whether exposure is assessed using HBM, exposure modeling, or another approach. Although our data allows a differentiated exposure assessment for cHMS and tHMS, the observed differences in hazard quotients (i.e., the ratio of the biomarker concentrations and their respective health-based guidance value) is only the result of differences in cHMS and tHMS uptake between the oral and dermal pathways. Our findings do not allow differentiation between risks associated with potentially different toxicological profiles of cHMS and tHMS. Thus, they also do not provide sufficient basis for recommending the use of diastereomerically pure *trans*-HMS in sunscreens, even if such use were technically feasible. Risk assessment can only be performed based on the current toxicological knowledge and the individual contributions of tHMS and cHMS to the overall toxicity of HMS remain unknown.

The pooled nature of the investigated urine samples precludes exposure or risk assessments on an individual level. However, representing approximately 18,700 individuals (100 per pool), these pooled urines are a valuable tool in identifying chemicals which are important contributors to population exposure and might be relevant risk drivers. If UV filter biomarker concentrations in a pooled urine sample exceed HBM guidance values, this reflects exceedances of these values among many contributing individuals, larger exceedances among fewer individuals, or anything in between. Either would indicate a need for exposure reduction, which can be achieved by stricter regulation of the respective UV filter in sunscreens.

The Australian population is particularly at risk of skin cancer; however, efficient sun protection is relevant all over the world. While the recommended primary protective measures are sun avoidance and wearing appropriate clothing, the application of sunscreen still plays an essential role in reducing UV radiation exposure. Accordingly, the results of this study must be viewed in a nuanced way, considering both the need for effective UV protection and the requirement for safe UV filter exposures.

The mere fact that exposure to UV filters occurred at all should not be regarded critically. On the contrary, the extraordinarily high detection frequencies for the investigated UV filters, with concentrations exceeding background exposures measured in Germany by one (adolescents/adults) to two (children) orders of magnitude, suggest a high compliance with sunscreen recommendations particularly among children, who have an even greater need for UV protection compared to adults. Notably, concentrations of the octocrylene biomarker CPAA were one order of magnitude below the HBM-I value. Although it cannot be excluded that some individuals exceeded the HBM-I value (since pooled urines were analyzed), there are no indications of OC-related health risks on a population level.

However, the vast number of pooled children’s urine samples exceeding the HBM-I value of HMS-CA5, and about 50% also exceeding the HBM-II value, suggests that homosalate exposure levels may exceed those considered acceptable and, in part, warrant measures to reduce exposure. Although the studied population was not representative for the Australian population, comparable exposures are likely for the entire population. Despite some uncertainty in risk assessment due to unknown differential contributions of cHMS and tHMS to total toxicity, we see need for action not only to directly mitigate potential health risks from HMS exposure but also to prevent loss of confidence in the safety of sunscreen products, which could have disastrous consequences by leading to ineffective sun protection behavior. Therefore, the maximum permitted concentration of homosalate in sunscreens should be regulated more strictly, potentially with a focus on products intended for children. Stricter regulations of HMS use are already in effect in the European Union and ASEAN and have been proposed by the Australian Therapeutic Goods Administration and the Australian Industrial Chemicals Introduction Scheme.

## Supplementary Material



## Data Availability

As all samples were deidentified and pooled in the pathology laboratory before analysis, any personal and identifiable information is removed, therefore a consent protocol is not required. The ap-proval for the ethics of this study was granted by The University of Queensland Medical Research Ethics Committee (2013000317). As per the ethics committee guidelines, all samples and obtained analytical results were stored in a secured facility and privately held database.

## Notes

Investigated biomarkers, further information on the analytical methods (instrument settings, validation, QC), metabolite correlations, biomarker concentrations by age groups and sampling periods, additional information on statistical models, implications for risk management—HMS-CA5 in comparison to tHMS-CA. (PDF)

## Abbreviations

3OH-cHMS: (1*R*,3*R*)/(1*S*,3*S*)-3-Hydroxy-3,5,5-trimethylcyclohexyl 2-hydroxybenzoate
3OH-tHMS: (1*R*,3*S*)/(1*S*,3*R*)-3-Hydroxy-3,5,5-trimethylcyclohexyl 2-hydroxybenzoate
5cx-EPS: 5-(((2-hydroxybenzoyl)­oxy) methyl)­heptanoic acid
5OH-EHS: 2-ethyl-5-hydroxyhexyl 2-hydroxybenzoate
ASEAN: Association of Southeast Asian Nations
cHMS: *cis*-homosalate
CPAA: 2-cyano-3,3-diphenylacrylic acid
DOCCA: 2-(carboxymethyl)­butyl 2-cyano-3,3-diphenyl acrylate; EHS 2-ethylhexyl salicylate
F_ue_: urinary excretion fraction
GM: geometric mean
HMS: homosalate
HMS-CA5: (1*R*,3*S*,5*S*)/(1*S*,3*R*,5*R*)-3-((2-hydroxybenzoyl)­oxy)-1,5-dimethylcyclohexane-1-carboxylic acid
OC: octocrylene
tHMS: *trans*-homosalate
tHMS-CA: (1*S*,5*R*)/(1*R*,5*S*)-5-((2-hydroxybenzoyl)­oxy)-3,3-dimethylcyclohexane-1-carboxylic acid
UV: ultraviolet
