# Supplementary material for: Assessing UV Filter Exposure in Queensland, Australia: Human Biomonitoring Evidence Highlighting Homosalate in Children
Source: Environ Sci Technol. 2026 Sep 4;60(37):26258–68. doi: 10.1021/acs.est.6c09445 (PMC13614191; doi:10.1021/acs.est.6c09445)
Supplement: Supplementary file 1 [file es6c09445_si_001.pdf]

Supporting Information for the Article:

Assessing UV Filter Exposure in Queensland, Australia: Human Biomonitoring Evidence Highlighting Homosalate in Children

Katharina E. Ebert[a], Danielle E. Que[b], Anne Lotz[a], Thomas Brüning[a], Leisa-Maree L. Toms[c], Peter Hobson[d], Heath Glover[d], Holger M. Koch[a,b], Jochen F. Mueller[b,e], Daniel Bury[a]*

[a] Institute for Prevention and Occupational Medicine of the German Social Accident Insurance, Institute of the Ruhr University Bochum (IPA), Bürkle-de-la-Camp-Platz 1, 44789 Bochum, Germany

[b] Queensland Alliance for Environmental Health Sciences (QAEHS), The University of Queensland, 20 Cornwall Street, Woolloongabba, Queensland 4102, Australia

[c] School of Public Health and Social Work, Faculty of Health, Queensland University of Technology, Kelvin Grove, Queensland 4059, Australia

[d] Sullivan Nicolaides Pathology, Bowen Hills, Queensland 4006, Australia

[e] Minderoo Centre – Plastics and Human Health, 20 Cornwall Street, Woolloongabba, Queensland 4102, Australia

***Corresponding Authors**

E-mail: daniel.bury@dguv.de
Tel.: +49 (0)30 13001 4414

E-mail: j.mueller@uq.edu.au
Tel.: +61 (0)410 661471

Table of contents

S1. Investigated exposure biomarkers 3

S2. Further information on the analytical methods 4
S2.1 General information 4
S2.2 OC method 6
S2.3 EHS/HMS method 8
S2.4 Method validation and quality control 10
S2.5 Validation results 11
S2.5.1. OC method 11
S2.5.2. EHS/HMS method 12

S3. Metabolite correlations 14

S4. Biomarker concentrations in pooled urine samples by age groups and 2-year sampling periods 16

S5. Additional information on the statistic model 18

S6. Implications for risk management – HMS-CA5 in comparison to tHMS-CA 23

S7. References 24

S1. Investigated exposure biomarkers

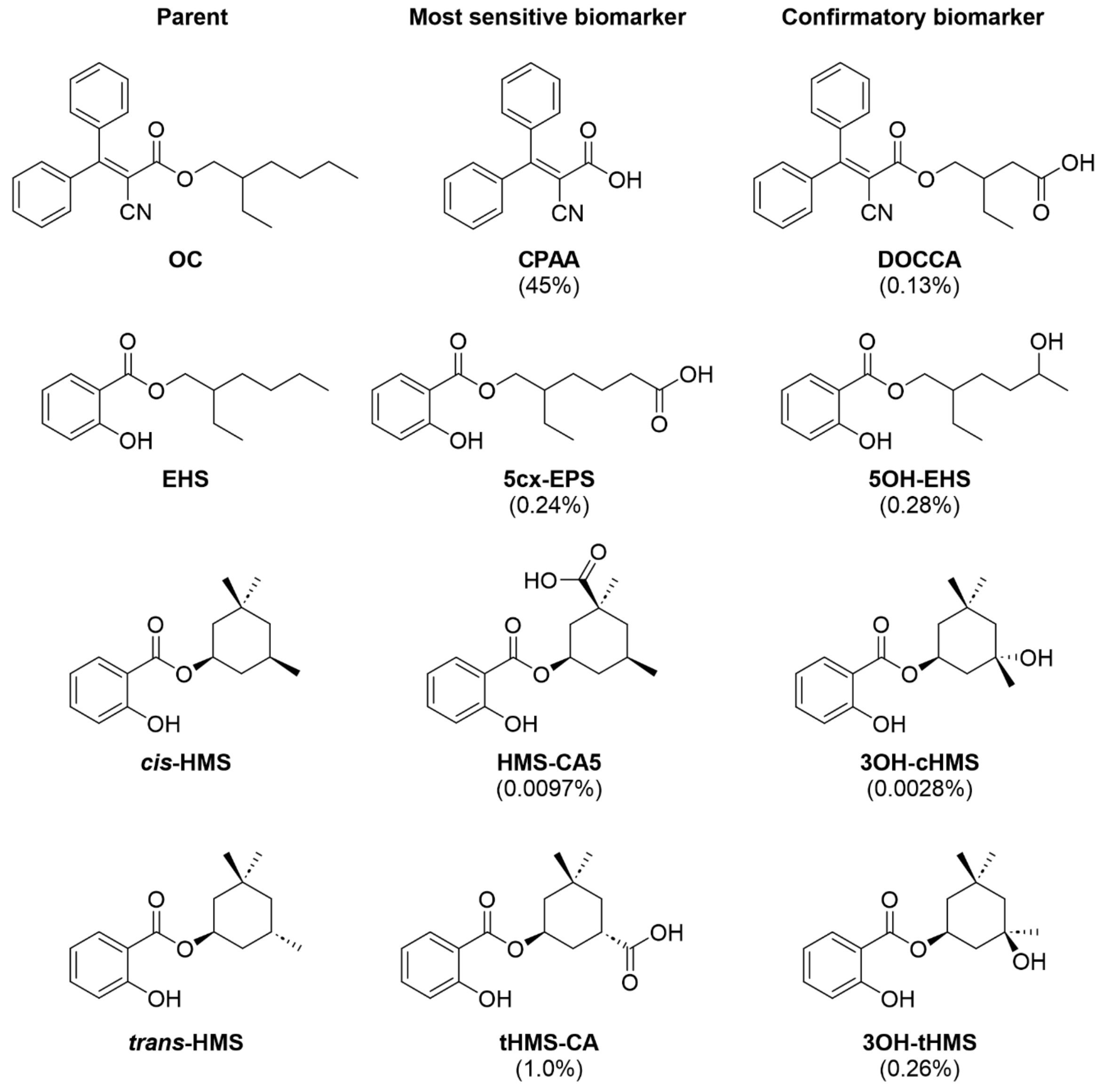

Figure S1. Chemical structures of the investigated UV filters and their most sensitive and confirmatory biomarkers. For simplicity, only one enantiomer of *cis*- and *trans*-HMS and their metabolites is shown. The urinary excretion fractions (F_{UE}) of the biomarkers are shown in brackets.[1–4]

S2. Further information on the analytical methods

S2.1 General information

Further chemicals and reagents

Analytical standards of CPAA, DOCCA, 5cx-EPS, 5OH-EHS, tHMS-CA, 3OH-tHMS, HMS-CA5, 3OH-cHMS, and the internal standards CPAA-d_{10}, DOCCA-d_{10}, 5cx-EPS-d_4, 5OH-EHS-d_4, tHMS-CA-d_4, and cHMS-CA-d_4 were synthesized at the Max Planck Institute for Multidisciplinary Sciences, Göttingen, Germany.[5–8] Acetonitrile, methanol, and water CHROMASOLV™ LC-MS grade were purchased from Honeywell Riedel-de Haën (Seelze, Germany). LiChropur® acetic acid 100% for LC-MS, LiChropur® formic acid 98%–100% for LC-MS and ammonium acetate BioXtra (≥ 98%) were obtained from Merck (Darmstadt, Germany), formic acid (AnalaR NORMAPUR® ACS, Reag. Ph. Eur.) from VWR (Darmstadt, Germany). β-Glucuronidase from E. coli K12 (aryl sulfatase-free, ≥ 140 u mL^{-1} at 37 °C according to manufacturer) was purchased from Roche Diagnostics (Mannheim, Germany).

Instrument setup

The Agilent 1260 Infinity II HPLC system (Agilent Technologies, Waldbronn, Germany), used for online sample cleanup and chromatographic separation, comprised a G7167A thermostated autosampler, two G7112B binary high-pressure gradient pumps (used as loading pump and LC pump, respectively) and a G7116A thermostated multi-column compartment with a six-port switching valve used as shown in Figure S2. Gradient delay volumes were 885–1085 µL for the loading pump flow path (pump and autosampler: 600–800 and 265 µL) and 600–800 µL for the LC pump flow path. An inline filter (porosity 0.5 µm; 3.0 mm diameter; Phenomenex, Aschaffenburg, Germany) was placed between autosampler and column compartment to protect the system from particles. Autosampler and column compartment were kept at 6 °C and 25 ±1 °C, respectively. An automatic needle wash with methanol/water 80:20 (v/v) was performed for 10 s after sample aspiration.

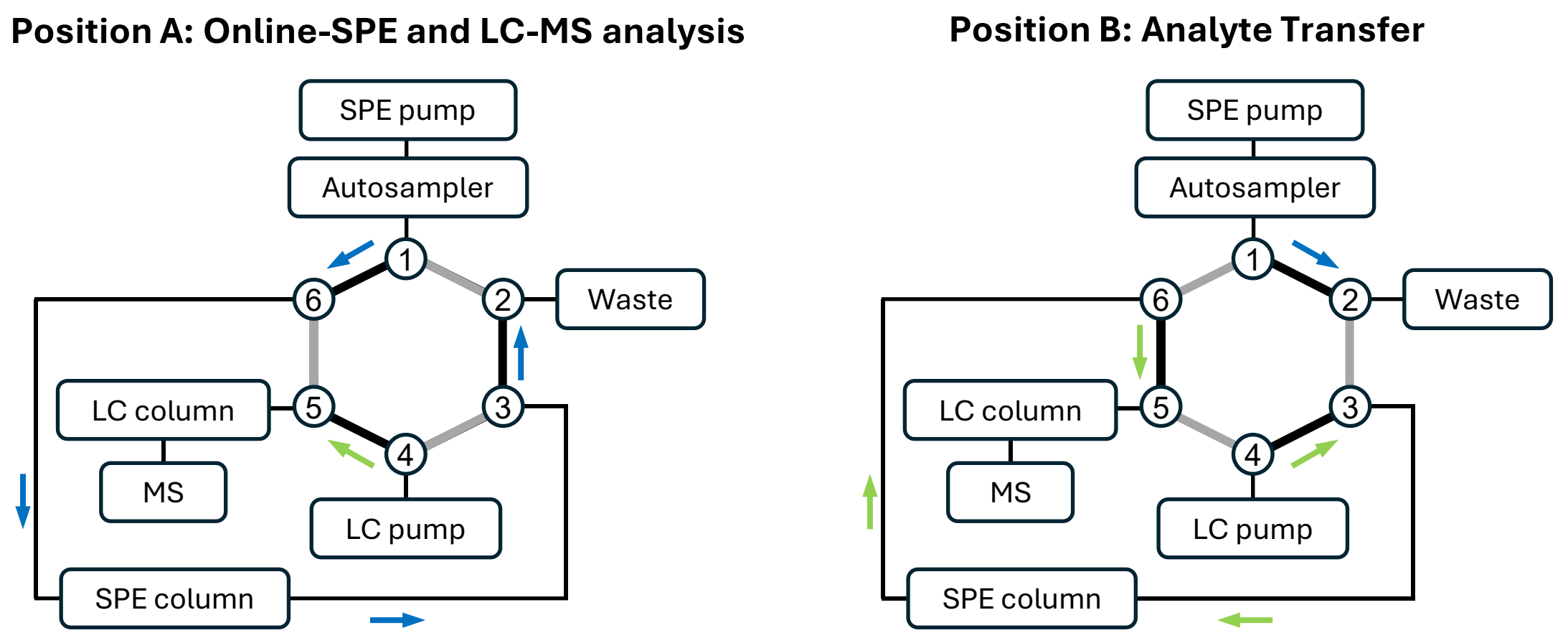

Figure S2. Instrument setup and flow paths used for online-SPE-LC-MS/MS. Connected ports are indicated by bold black lines. Blue arrows show the flow path of the loading pump, green arrows the flow path of the LC pump. See the respective method description for the valve switching times.

Standard Stock Solutions

Standard stock solutions (~1 g L^{-1}) of the non-labeled analytes were prepared by dissolving approximately 10 mg, weighed exactly in a 10 mL volumetric flask, in acetonitrile. After preparation of appropriate intermediate dilutions, three mixed stock solutions were prepared by serial dilution with acetonitrile, containing 5000 µg L^{-1}, 250 µg L^{-1} and 20 µg L^{-1} (Mix 1, Mix 2, Mix 3) of CPAA, 500 µg L^{-1}, 25 µg L^{-1} and 2 µg L^{-1} of 5OH-EHS, and 250 µg L^{-1}, 12.5 µg L^{-1} and 1 µg L^{-1} each of DOCCA, 5cx-EPS, *t*HMS-CA, 3OH-*t*HMS, HMS-CA5 and 3OH-*c*HMS.

Stock solutions of deuterium-labeled internal standards in acetonitrile were prepared in volumetric flasks and further diluted in acetonitrile. The dilutions were used to prepare an internal standard mix (200 µg L^{-1} CPAA-d_{10}, 20 µg L^{-1} 5OH-EHS-d_4, 10 µg L^{-1} DOCCA-d_{10} and 5cx-EPS-d_4, 5 µg L^{-1} tHMS-CA-d_4 and cHMS-CA-d_4).

All these solutions were stored in glass vials with silicone/polytetrafluoroethylene (PTFE)-sealed screw caps at −20 °C.

Calibration

Joint calibration solutions were prepared for the two analytical methods (OC and EHS/HMS).

Seven calibration solutions (concentration range 0.2 µg L^{-1} to 100 µg L^{-1} CPAA, 0.02 µg L^{-1} to 10 µg L^{-1} 5OH-EHS, and 0.01 µg L^{-1} to 5 µg L^{-1} for the remaining analytes) were prepared by diluting the abovementioned mixed stock solutions with LC−MS grade water. The acetonitrile content of each calibration solution did not exceed 2%. 300 µL aliquots of each calibration solution as well as LC-MS grade water as blank were transferred into silanized 1.5 mL HPLC vials with silicone/PTFE screw caps (N9, Macherey-Nagel, Düren, Germany) immediately after preparation and stored at −20 °C. During sample preparation, calibration samples were treated identically to urine samples.

Calibration functions were calculated by weighted (concentration^{-1}) linear regression of the peak area ratio (standard/internal standard) as a function of the analyte concentration. *c*HMS-CA-d_4 was used as a surrogate internal standard for HMS-CA5, 3OH-*t*HMS and 3OH-*c*HMS.

Preparation of quality control material

Joint quality control material was prepared for the two analytical methods (OC and EHS/HMS).

Three quality control (QC) materials (Q_{low}, Q_{med}, Q_{high}) were prepared by mixing different urine samples with native concentrations of the relevant OC, EHS, and HMS metabolite biomarkers. Native DOCCA, 3OH-tHMS, HMS-CA5 and 3OH-*c*HMS concentrations were too low for adequate coverage of the calibration range and were additionally spiked using the respective standard dilutions. The acetonitrile content of the quality control material did not exceed 0.3%. The pooled urine samples were frozen, thawed, and filtered three times to obtain homogenous sample material and were subsequently aliquoted (300 µL) into silanized 1.5 mL screw cap vials. The quality control material was stored at −20 °C until further use.

S2.2 OC method

In the previously published method[9], OC biomarkers were detected in a single method together with EHS biomarkers. However, recently, increasing batch-to-batch variation of the used analytical column, resulting in pronounced sample-matrix-dependent shifting in retention times for CPAA and in unresolved matrix interferences in some urine samples, affected the robustness of chromatographic separation for CPAA. Hence, a new method had to be developed. While previous studies have also investigated the minor OC biomarker 5OH-OC, its relatively low sensitivity led us to exclude it from further measurements.

LC conditions

A TurboFlow™ C8 column (0.5×50 mm; Thermo Fisher Scientific, Franklin, MA, USA) was used for matrix depletion and analyte enrichment and a Hypersil GOLD™ (C18) column (150×3 mm; particle size 3 µm; with corresponding Uniguard™ Direct-Connection Guard Cartridge Holder and Hypersil GOLD Defender™ Guard Cartridge; Thermo Scientific, Franklin, MA, USA) was used for analyte separation. Injection volume was 100 µL.

Eluents were water with 0.05% formic acid (A) and 95:5 acetonitrile:water with 0.05% formic acid (B). LC pump gradients are shown in Table S1.

Table S1. LC pump gradients of the OC analytical method

Loading pump			Analytical pump		
**Time [min]**	**% B**	**Flow [µL/min]**	**Time [min]**	**% B**	**Flow [µL/min]**
0	5	1500	0	30	400
3	5	1500	4	30	400
3.1	5	200	20.5	39.5	400
6	5	200	22	55	400
7	5	500	33	60.5	400
10	100	500	33.5	100	400
21	100	500	38.5	100	400
22	5	200	39	30	400
46	5	200	49	30	400
47	5	1500			
49	5	1500			

Valve switching times were 0 (load/re-equilibrate position), 3 (transfer position), 9 min (load/re-equilibrate position) (see Figure S2).

MS conditions

Detection of OC metabolites was performed with electrospray-tandem mass spectrometry (ESI-MS/MS) using a 5500 triple quadrupole mass spectrometer (Sciex, Darmstadt, Germany) in time-programmed multiple reaction monitoring (*scheduled* MRM™) mode with polarity switching. The instrument gases (nitrogen) were set to: curtain gas 40 psi, nebulizer gas (gas 1) 40 psi, heater gas (gas 2) 50 psi, collision gas (CAD) 8 arbitrary units. Source heater temperature was 450 °C. Ion spray voltage, entrance potential and cell exit potential for positive (negative) ion mode were set to 5.5 kV, 10 V, and 12 V (−4.5 kV, −10 V and −10 V), respectively. See Table S2 for further MRM parameters.

Like the previously published modified method[9] and unlike the original method[6], for CPAA the herein described method initially used the mass transitions *m/z* 248->203 and *m/z* 248->202 as quantifier and qualifier transitions, respectively. However, this qualifier showed insufficiently low sensitivity and issues with matrix interferences at lower concentrations. Hence, the mass transition *m/z* 249->204 of the ^{13}C satellite (corresponding to the quantifier mass transition) was introduced as a more sensitive and more rugged qualifier transition, although the *m/z* 248->202 transition remained in the method.

Table S2. Further MS parameters and MRM transitions for the OC analytical method

Analyte	Type	ESI mode	t_R [min]	t_R tolerance [s]	Parent m/z	Fragment m/z	Dwell time [ms]	DP [V]	CE [eV]
CPAA	Quan	Negative	20.2	90	248	203	100	−55	−28
	Qual1	Negative	20.2	90	249	204	30	−55	−18
	Qual2	Negative	20.2	90	248	202	30	−55	−23
CPAA-d_{10}	Quan	Negative	19.8	90	258	214	100	−45	−11
	Qual	Negative	19.8	90	258	212	30	−45	−21
DOCCA	Quan	Positive	29.2	30	364	250	50	50	12
	Qual	Positive	29.2	30	364	115	15	50	15
DOCCA-d_{10}	Quan	Positive	29.0	30	374	260	50	50	12
	Qual	Positive	29.0	30	374	115	15	50	17

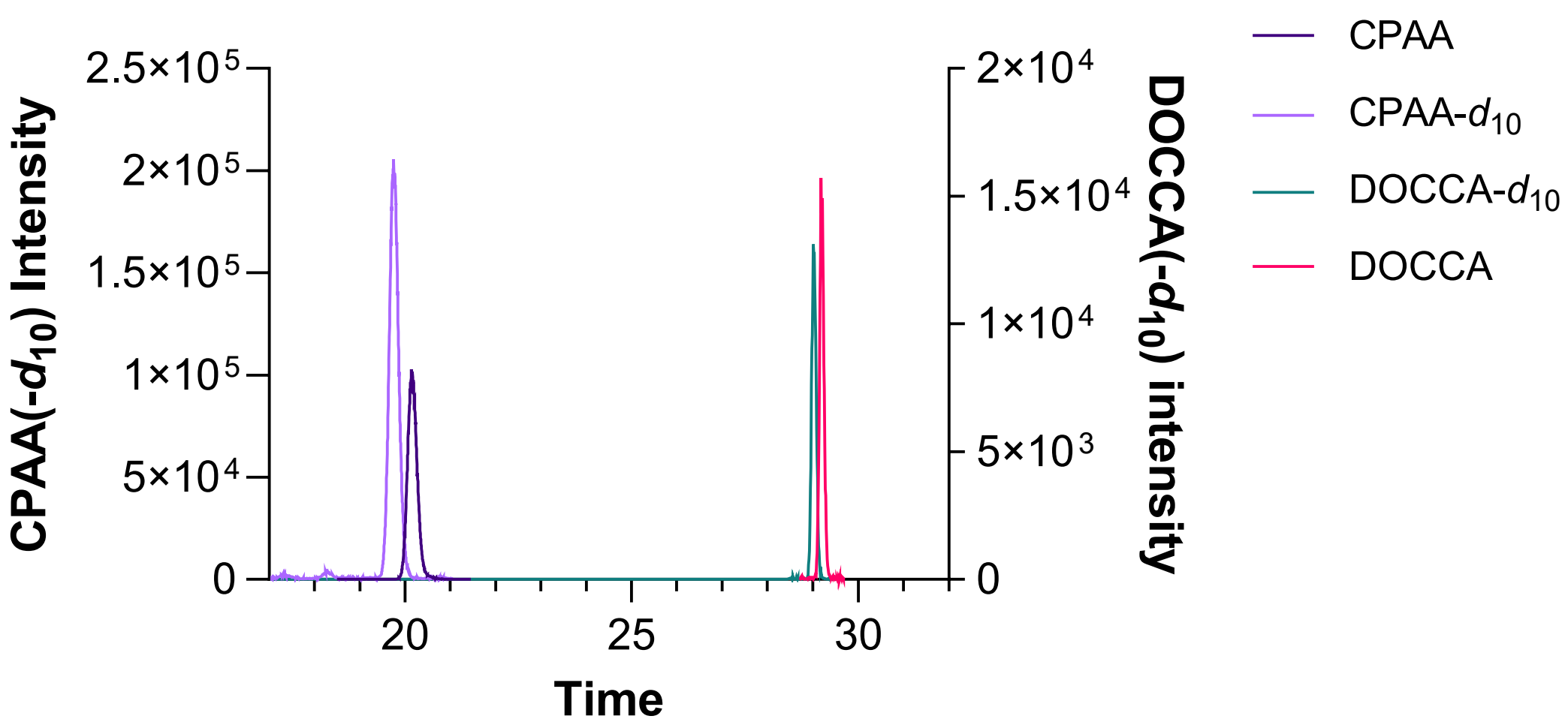

Figure S3. Exemplary LC-MS/MS chromatogram of the OC method, showing one pooled urine sample of the herein described study, containing 73 µg L^{-1} CPAA and 0.94 µg L^{-1} DOCCA. Quantifier mass transitions of both analytes and their authentic internal standards are shown.

S2.3 EHS/HMS method

While previous studies have also investigated the minor EHS biomarker 5oxo-EHS, its relatively low sensitivity led us to exclude it from further measurements.

LC conditions

The herein described EHS/HMS method is based on a previously published HMS method [7]. A TurboFlow™ C8 column (0.5×50 mm; Thermo Fisher Scientific, Franklin, MA, USA) was used for matrix depletion and analyte enrichment and an Accucore™ C18 column (150×3 mm; particle size 2.6 µm; with corresponding Uniguard™ Direct-Connection Guard Cartridge Holder and Accucore™ C18 Defender™ Guard Cartridge; Thermo Scientific, Franklin, MA, USA) was used for analyte separation. Injection volume was 100 µL.

Eluents were water with 0.05% acetic acid (A) and 95:5 acetonitrile:water with 0.05% acetic acid (B). LC pump gradients are shown in Table S3.

Table S3. LC pump gradients of the EHS/HMS analytical method

Loading pump			Analytical pump		
**Time [min]**	**% B**	**Flow [µL/min]**	**Time [min]**	**% B**	**Flow [µL/min]**
0	5	1500	0	35	300
3	5	1500	2.9	35	300
3.1	5	200	4.4	53	300
6	5	200	16.4	67	300
7	5	400	18.4	100	300
10	100	400	22.9	100	300
21	100	400	23.4	35	300
22	5	400	30	35	300
27	5	400			
28	5	1500			
30	5	1500			

Valve switching times were 0 (load/re-equilibrate position), 3 (transfer position), 6 (load/re-equilibrate position) min (see Figure S2).

MS conditions

Detection of EHS and HMS metabolites was performed with electrospray-tandem mass spectrometry (ESI-MS/MS) using a 5500 triple quadrupole mass spectrometer (Sciex, Darmstadt, Germany) in time-programmed multiple reaction monitoring (*scheduled* MRM™) mode with polarity switching. The instrument gases (nitrogen) were set to: curtain gas 40 psi, nebulizer gas (gas 1) 40 psi, heater gas (gas 2) 50 psi, collision gas (CAD) 8 arbitrary units. Source heater temperature was 450 °C. Ion spray voltage, entrance potential and cell exit potential for positive (negative) ion mode were set to 5.5 kV, 10 V, and 12 V (−4.5 kV, −10 V and −10 V), respectively. See Table S4 for further MRM parameters.

The relevant HMS-CA and 3OH-HMS isomers showed identical optimum MS parameters. Large MRM windows could therefore be chosen to record all HMS-CA and OH-HMS isomers in the same MRM window. cHMS-CA-d_4 served as surrogate internal standard for HMS-CA5, 3OH-tHMS, and 3OH-cHMS.

Table S4. Further MS parameters and MRM transitions for the EHS/HMS analytical method

Analyte	Type	ESI mode	t_R [min]	t_R tolerance [s]	Parent m/z	Fragment m/z	Dwell time [ms]	DP [V]	CE [eV]
5Cx-EPS	Quan	Negative	13.4	30	279	93	50	−60	−48
	Qual	Negative	13.4	30	279	137	10	−60	−19
5Cx-EPS-*d*4	Quan	Negative	13.3	30	283	97	50	−50	−49
	Qual	Negative	13.3	30	283	141	10	−50	−18
5OH-EHS	Quan	Positive	14.2	30	267	129	50	45	9
	Qual	Positive	14.2	30	267	111	10	45	16
5OH-EHS-*d*4	Quan	Positive	14.1	30	271	129	50	50	9
	Qual	Positive	14.1	30	271	125	10	50	36
HMS-CA	Quan	Negative	14.2[a]	90	291	137	40	−80	−22
	Qual	Negative	14.2[a]	90	291	93	10	−80	−54
HMS-CA-*d*4	Quan	Negative	14.2[b]	90	295	141	40	−80	−20
	Qual	Negative	14.2[b]	90	295	69	10	−80	−85
OH-HMS	Quan	Negative	17.2[c]	240	277	137	40	−120	−28
	Qual	Negative	17.2[c]	240	277	93	10	−120	−48

[a] MRM window includes multiple HMS-CA isomers, among them tHMS-CA (t_R=13.6 min) and HMS-CA5 (t_R=14.9 min)

[b] MRM window includes both tHMS-CA-*d*4 (t_R=13.5 min) and cHMS-CA-*d*4 (t_R=14.5 min)

[c] MRM window includes multiple OH-HMS isomers, among them 3OH-tHMS (t_R=14.8 min) and 3OH-cHMS (t_R=18.7 min)

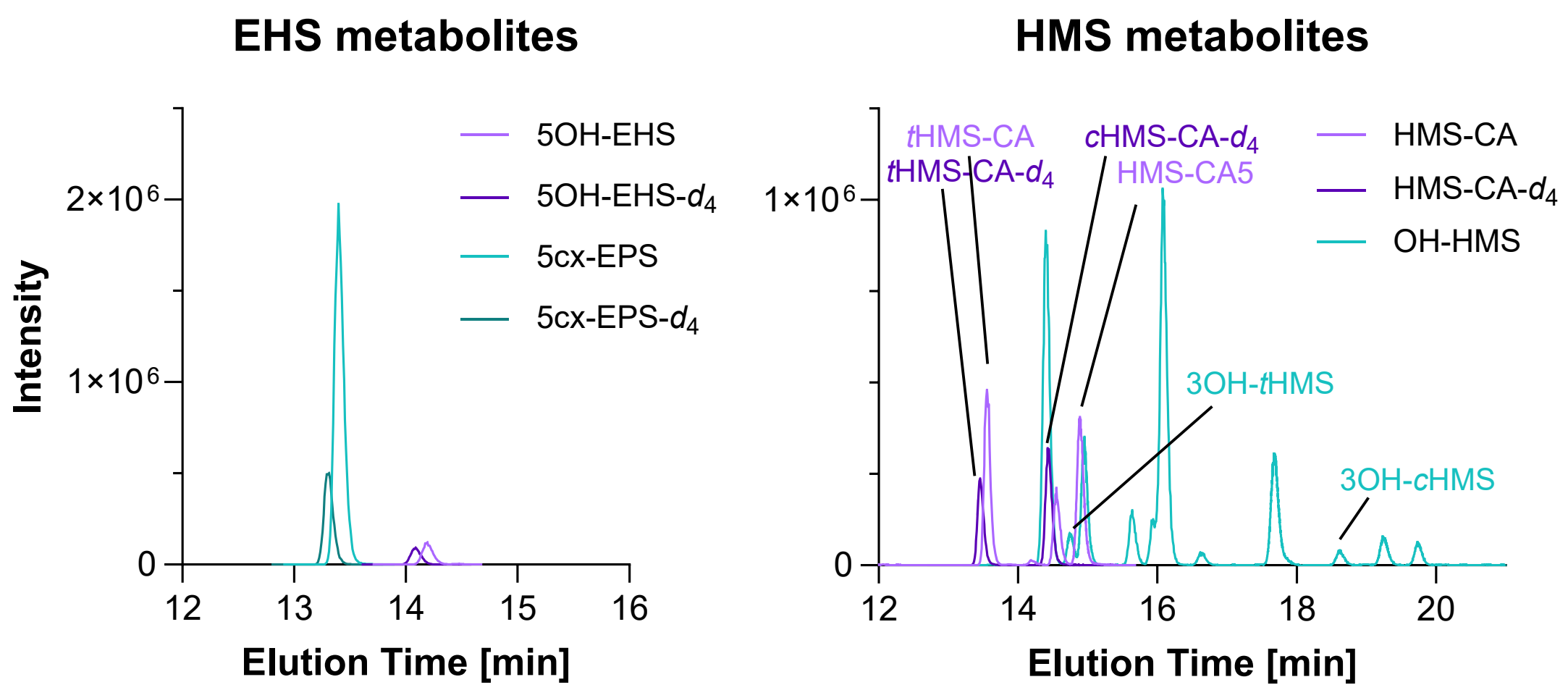

Figure S4. Exemplary LC-MS/MS chromatogram of the EHS/HMS method, showing one pooled urine sample of the herein described study, containing 4 µg L^{-1} 5cx-EPS, 2.2 µg L^{-1} 5OH-EHS, 1.3 µg L^{-1} tHMS-CA, 0.21 µg L^{-1} 3OH-tHMS, 0.68 µg L^{-1} HMS-CA5 and 0.28 µg L^{-1} 3OH-cHMS. Quantifier mass transitions of analytes and the internal standards are shown. The additional unlabeled peaks in the HMS metabolite chromatogram are known isomer peaks of the respective biomarkers (described in detail in Ebert et al. (2022)[4]).

S2.4 Method validation and quality control

Limits of quantification (LOQ) were estimated based on a signal-to-noise ratio of 10 in matrix as described in Bury et al., (2018)[6].

Ion ratios between the quantifier and qualifier mass transitions were determined for all analyte peaks and compared to the average peak area ratio of the calibration samples. The maximum permitted tolerances were chosen according to decision 2002/657/EC [10] and were ±20% for all analytes apart from DOCCA, DOCCA-d_{10} (±25%), *t*HMS-CA-d_4, and *c*HMS-CA-d_4 (±50%).

Samples with unusual peaks (ion ratios, peak shapes, …) were reanalyzed using freshly prepared samples. If the calculated concentrations permitted, diluted urine samples were used for reanalysis in order to reduce possible matrix interferences.

Enzymatic deconjugation was already shown to be complete after less than 3 h for all analytes in previous publications [5,6,8]. As the sample preparation was identical to these validated methods, no further experiments were performed apart from confirming that identical analyte concentrations were obtained when measuring QC samples with both the old and new analytical methods.

Analyte stability in urine was evaluated based on previous quality control charts in our lab; no analyte degradation was observed over a span of three years during which identical QC materials were used. This is in agreement with recent results showing that (native and predominantly conjugated) 5cx-EPS and 5OH-EHS remain stable in urine samples stored at −20 °C for at least 8 years (manuscript in preparation).

To determine the method imprecision, quality control material was analyzed eight times within one analytical batch (intra-day imprecision) and on eight different days (inter-day imprecision). After validation, this material was further analyzed in each batch for continuous quality control (quality control charts).

The method accuracy and the influence of matrix effects were determined by analyzing 17 different urine samples (0.28 to 2.8 g L^{-1} creatinine), without spiking and spiked at three different concentration levels before sample preparation. Native analyte concentrations were subtracted from the spiked samples' concentrations and relative recoveries were calculated. For spiking the urine samples, 1 M ammonium acetate buffer (pH = 6.0–6.4) was prepared containing three times the desired final concentration in urine (urine/buffer ratio during sample preparation = 3) using a mixed analyte solution in acetonitrile. The acetonitrile content of the spiked buffers was 2%. Sample preparation was performed as previously described.

S2.5 Validation results

S2.5.1. OC method

LOQs in urine were 0.5 µg L^{-1} for CPAA and 0.1 µg L^{-1} for DOCCA. Compared to the previously published method [9], the LOQ for DOCCA increased by a factor of two due to lower sensitivity of DOCCA when using formic acid instead of acetic acid as eluent additive. However, as DOCCA is only a confirmatory biomarker relevant for confirming higher exposures, this was considered irrelevant from an exposure and risk assessment perspective. The LOQ of the main exposure biomarker (CPAA) remained unchanged.

Table S5. Method imprecisions of the OC method

		Intra-day imprecision (n = 8)		Inter-day imprecision (n = 8)	
**Metabolite**		**Mean conc. (range) [µg L^{-1}]**	**CV**	**Mean conc. (range) [µg L^{-1}]**	**CV**
CPAA	Q_{low}	2.22 (2.13–2.30)	2.4%	2.09 (2.01–2.15)	3.8%
	Q_{med}	41.7 (40.9–42.5)	1.6%	41.0 (39.6–42.9)	2.8%
	Q_{high}	84.3 (83.3–85.9)	0.9%	83.1 (78.9–86.5)	2.8%
DOCCA	Q_{low}	0.15 (0.13–0.17)	6.9%	0.15 (0.13–0.16)	7.6%
	Q_{med}	0.87 (0.81–0.93)	4.9%	0.88 (0.83–0.95)	6.7%
	Q_{high}	2.52 (2.43–2.70)	3.0%	2.53 (2.41–2.72)	5.3%

Table S6. Relative recoveries of OC metabolite standards in spiked urine samples[a]

**Metabolite**	c_{native} **[µg L^{-1}]**	c_{spiked} **[µg L^{-1}]**	$c_{measured} - c_{native}$ **(range) [µg L^{-1}]**	**Mean RR (range) [%]**
CPAA	<LOQ–13	5	5.17 (4.29–5.82)	103% (86–116%)
		25	24.6 (21.7–27.1)	99% (87–108%)
		60	59.1 (51.7–62.4)	98% (86–104%)
DOCCA	<LOQ–0.25	0.60	0.66 (0.58–0.77)	110% (97–128%)
		3	3.19 (2.93–3.80)	106% (98–127%)
		4	4.29 (3.84–5.10)[b]	107% (96–128%)

[a] n = 16/17; 0.28 to 2.8 g L^{-1} creatinine; for CPAA, 1 out of 17 samples was excluded as it did not conform to quality control criteria (deformation of the internal standard peak)

c_{native}: native concentration; c_{spiked}: spiked concentration, $c_{measured}$: measured concentration, RR: relative recovery

[b] maximum value was slightly outside of the calibration range (by 0.1 µg/L)

S2.5.2. EHS/HMS method

LOQs in urine were 0.1 µg L^{-1} for 5OH-EHS, 0.04 µg L^{-1} for 3OH-cHMS, 0.03 µg L^{-1} for tHMS-CA, and 0.02 µg L^{-1} for 5cx-EPS, 3OH-tHMS, and HMS-CA5. Compared to the previously published method[9], the LOQ for 5OH-EHS increased by a factor of two. However, as 5OH-EHS is only a confirmatory biomarker relevant for confirming higher exposures, this was considered irrelevant from an exposure and risk assessment perspective. The LOQ of the main EHS exposure biomarker (5cx-EPS) as well as the HMS biomarkers remained unchanged.

Table S7. Method imprecisions of EHS/HMS method

		**Intra-day imprecision (n = 8)**		**Inter-day imprecision (n = 8)**	
**Metabolite**		**Mean conc. (range) [µg L^{-1}]**	**CV**	**Mean conc. (range) [µg L^{-1}]**	**CV**
5cx-EPS	Q_{low}	0.104 (0.099–0.107)	2.8%	0.106 (0.103–0.118)	4.7%
	Q_{med}	1.23 (1.18–1.27)	2.7%	1.29 (1.23–1.41)	4.4%
	Q_{high}	4.21 (4.04–4.42)	2.7%	4.38 (4.21–4.90)	5.0%
5OH-EHS	Q_{low}	0.17 (0.16–0.17)	2.3%	0.17 (0.16–0.18)	3.6%
	Q_{med}	1.29 (1.28–1.32)	1.4%	1.30 (1.26–1.34)	2.4%
	Q_{high}	4.35 (4.29–4.46)	1.3%	4.35 (4.24–4.48)	1.8%
*t*HMS-CA	Q_{low}	0.116 (0.110–0.121)	3.2%	0.116 (0.112–0.120)	2.2%
	Q_{med}	0.929 (0.893–0.976)	3.3%	0.962 (0.929–1.00)	2.7%
	Q_{high}	2.67 (2.61–2.71)	1.3%	2.78 (2.67–2.87)	2.7%
3OH-*t*HMS	Q_{low}	0.072 (0.070–0.074)	1.7%	0.074 (0.070–0.077)	2.7%
	Q_{med}	0.485 (0.476–0.498)	1.4%	0.487 (0.460–0.504)	3.1%
	Q_{high}	2.24 (2.20–2.30)	1.8%	2.32 (2.19–2.44)	2.8%
HMS-CA5	Q_{low}	0.056 (0.053–0.058)	2.9%	0.058 (0.056–0.060)	2.3%
	Q_{med}	0.385 (0.376–0.392)	1.4%	0.398 (0.385–0.406)	2.0%
	Q_{high}	1.98 (1.95–2.03)	1.5%	2.09 (1.98–2.14)	2.7%
3OH-*c*HMS	Q_{low}	0.125 (0.122–0.128)	1.9%	0.127 (0.125–0.134)	4.0%
	Q_{med}	0.468 (0.448–0.483)	2.5%	0.474 (0.425–0.506)	5.1%
	Q_{high}	2.83 (2.71–2.92)	2.5%	2.96 (2.74–3.22)	5.0%

Table S8. Relative recoveries of EHS/HMS metabolite standards in spiked urine samples[a]

Metabolite	c_{native} [µg L^{-1}]	c_{spiked} [µg L^{-1}]	$c_{measured} - c_{native}$ (range) [µg L^{-1}]	Mean RR (range) [%]
5cx-EPS	<LOQ–0.296	0.10	0.108 (0.096–0.121)	108% (96–121%)
		0.50	0.530 (0.469–0.556)	106% (94–111%)
		4.0	4.03 (3.78–4.27)	101% (94–107%)
5OH-EHS	<LOQ–0.22	0.50	0.50 (0.38–0.58)	100% (77–115%)
		2.5	2.37 (2.02–2.68)	95% (81–107%)
		8.0	7.47 (6.29–8.34)	93% (79–104%)
tHMS-CA	<LOQ–0.11	0.1	0.098 (0.087–0.108)	98% (87–108%)
		0.5	0.477 (0.430–0.521)	95% (86–104%)
		4	3.66 (3.28–3.99)	92% (82–100%)
3OH-tHMS	<LOQ	0.10	0.096 (0.082–0.109)	96% (82–109%)
		0.50	0.447 (0.382–0.483)	89% (76–97%)
		4.0	3.35 (2.86–3.57)	84% (72–89%)
HMS-CA5	<LOQ–0.021	0.10	0.105 (0.089–0.124)	105% (89–124%)
		0.50	0.520 (0.428–0.564)	104% (86–113%)
		4.0	3.97 (3.47–4.17)	99% (87–104%)
3OH-cHMS	<LOQ	0.10	0.097 (0.078–0.113)	97% (78–113%)
		0.50	0.462 (0.375–0.506)	92% (75–101%)
		4.0	3.59 (2.93–3.99)	90% (73–100%)

[a] n = 17; 0.28 to 2.8 g L^{-1} creatinine; c_{native}: native concentration; c_{spiked}: spiked concentration, $c_{measured}$: measured concentration, RR: relative recovery

S3. Metabolite correlations

Spearman's rho coefficients were calculated using SPSS (Analyze->Correlate->Bivariate). Concentrations below the respective LOQ were set to LOQ/2. In addition to correlations between main and confirmatory biomarkers of each investigated UV filter, correlations were also calculated for biomarkers of different UV filters (see Figure S5). See Figure S6 for the plotted metabolite correlations main/confirmatory biomarker pairs of the investigated UV filters.

Correlations were excellent for all main/confirmatory biomarker pairs, with Spearman's rho >0.84 for all pairs. However, even between biomarkers of different parent UV filters, correlations were still good with correlations coefficients >0.5. As the main exposure source to UV filters is the application of sunscreen and sunscreens typically contain multiple UV filters, this correlation is not unexpected.

This is the first population study with sufficient detection frequencies for correlation analysis of the HMS biomarkers. In the case of the OC and EHS metabolites, the metabolite ratios were in reasonable agreement with previous studies.

Interestingly, in the case of the EHS biomarkers, different metabolite ratios were observed for children (≤15 years) and adolescents/adults (≥16 years), with roughly a factor of 2 higher 5cx-EPS concentrations in children (Figure S6). As 5cx-EPS is the main biomarker used for daily intake calculation and risk assessment using the HBM-I value, this discrepancy in metabolite ratios results in a more conservative risk assessment in children, which is favorable due to them being a particularly vulnerable population.

Correlations

			CPAA	DOCCA	5OH-EHS	5cx-EPS	tHMS-CA	3OH-tHMS	HMS-CA5	3OH-cHMS
Spearman's rho	CPAA	Correlation Coefficient	--							
		Sig. (2-tailed)	.							
		N	187							
	DOCCA	Correlation Coefficient	.891**	--						
		Sig. (2-tailed)	<.001	.						
		N	187	187						
	5OH-EHS	Correlation Coefficient	.705**	.577**	--					
		Sig. (2-tailed)	<.001	<.001	.					
		N	187	187	187					
	5cx-EPS	Correlation Coefficient	.794**	.688**	.927**	--				
		Sig. (2-tailed)	<.001	<.001	<.001	.				
		N	187	187	187	187				
	tHMS-CA	Correlation Coefficient	.842**	.779**	.763**	.762**	--			
		Sig. (2-tailed)	<.001	<.001	<.001	<.001	.			
		N	187	187	187	187	187			
	3OH-tHMS	Correlation Coefficient	.892**	.832**	.756**	.788**	.953**	--		
		Sig. (2-tailed)	<.001	<.001	<.001	<.001	<.001	.		
		N	187	187	187	187	187	187		
	HMS-CA5	Correlation Coefficient	.927**	.900**	.720**	.810**	.883**	.928**	--	
		Sig. (2-tailed)	<.001	<.001	<.001	<.001	<.001	<.001	.	
		N	187	187	187	187	187	187	187	
	3OH-cHMS	Correlation Coefficient	.786**	.771**	.753**	.812**	.828**	.849**	.847**	--
		Sig. (2-tailed)	<.001	<.001	<.001	<.001	<.001	<.001	<.001	.
		N	187	187	187	187	187	187	187	187

**. Correlation is significant at the 0.01 level (2-tailed).

Figure S5. Spearman rank correlation coefficients for all investigated biomarkers.

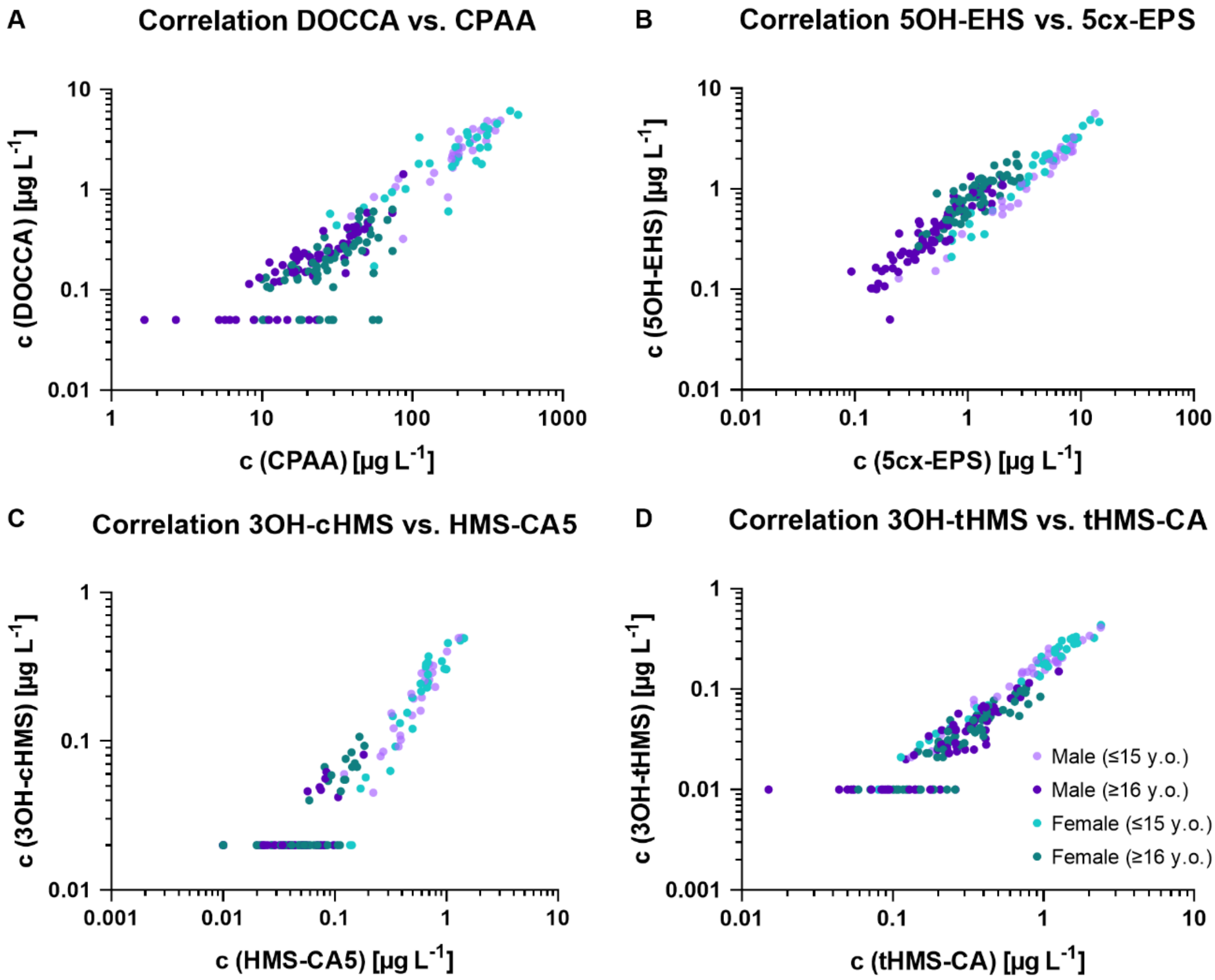

Figure S6. Metabolite correlations between main and confirmatory biomarkers for each of the investigated UV filters. Different colors are used for children (≤15 years) and adults (≥16 years) and males and females.

S4. Biomarker concentrations in pooled urine samples by age groups and 2-year sampling periods

Table S9. Concentrations of the main biomarkers CPAA (OC), 5cx-EPS (EHS), tHMS-CA (*trans*-HMS), and HMS-CA5 (*cis*-HMS) per sample collection period. Data was grouped according to age into 'children' (≤ 15 years) and 'adults' (≥ 16 years). For each year, the number of pooled urines (n) is given. As the investigated samples were pooled urine samples, it was decided not to calculate percentiles apart from the median.

Biomarker	Year	n	n>LOQ (%)	Min [µg L^{-1}]	Median [µg L^{-1}]	Mean$_{geometric}$ [µg L^{-1}]	Max [µg L^{-1}]
Children (≤15)		60					
CPAA	2012–2013	8	8 (100%)	31.3	69.2	58.5	89.8
	2014–2015	8	8 (100%)	26.7	97.2	83.6	240
	2016–2017	4	4 (100%)	55.5	129	103	173
	2018–2019	16	16 (100%)	110	222	234	503
	2020–2021	16	16 (100%)	111	218	222	355
	2022–2023	8	8 (100%)	203	308	299	384
5cx-EPS	2012–2013	8	8 (100%)	0.653	1.24	1.30	2.79
	2014–2015	8	8 (100%)	0.244	3.52	2.13	9.45
	2016–2017	4	4 (100%)	0.719	3.32	2.45	5.32
	2018–2019	16	16 (100%)	1.03	5.94	5.01	14.5
	2020–2021	16	16 (100%)	1.65	4.95	4.73	10.4
	2022–2023	8	8 (100%)	2.37	6.32	5.15	8.57
tHMS-CA	2012–2013	8	8 (100%)	0.080	0.201	0.196	0.344
	2014–2015	8	8 (100%)	0.100	0.505	0.379	1.26
	2016–2017	4	4 (100%)	0.113	0.621	0.429	0.966
	2018–2019	16	16 (100%)	0.361	1.04	1.03	2.40
	2020–2021	16	16 (100%)	0.492	1.21	1.10	1.64
	2022–2023	8	8 (100%)	0.716	1.39	1.36	2.39
HMS-CA5	2012–2013	8	8 (100%)	0.056	0.101	0.121	0.314
	2014–2015	8	8 (100%)	0.044	0.325	0.211	0.761
	2016–2017	4	4 (100%)	0.051	0.226	0.178	0.492
	2018–2019	16	16 (100%)	0.170	0.651	0.573	1.44
	2020–2021	16	16 (100%)	0.221	0.597	0.544	1.03
	2022–2023	8	8 (100%)	0.373	0.665	0.662	1.28

Table S9 cont.

Biomarker	Year	n	n>LOQ (%)	Min [µg L^{-1}]	Median [µg L^{-1}]	$Mean_{geometric}$ [µg L^{-1}]	Max [µg L^{-1}]
Adolescents/Adults (≥16)		127					
CPAA	2012–2013	16	16 (100%)	2.67	12.6	11.9	27.1
	2014–2015	16	16 (100%)	6.69	11.8	12.1	20.4
	2016–2017	16	16 (100%)	1.65	23.3	19.2	55.1
	2018–2019	31	31 (100%)	9.58	26.1	28.9	73.5
	2020–2021	32	32 (100%)	16.0	34.7	31.3	59.4
	2022–2023	16	16 (100%)	19.0	44.1	43.2	86.8
5cx-EPS	2012–2013	16	16 (100%)	0.139	0.700	0.587	2.79
	2014–2015	16	16 (100%)	0.204	0.529	0.574	1.96
	2016–2017	16	16 (100%)	0.093	0.854	0.643	2.21
	2018–2019	31	31 (100%)	0.266	0.901	0.860	2.57
	2020–2021	32	32 (100%)	0.207	0.720	0.731	2.70
	2022–2023	16	16 (100%)	0.218	0.841	0.809	2.87
tHMS-CA	2012–2013	16	15 (94%)	<LOQ [0.03]	0.123	<0.106	0.305
	2014–2015	16	16 (100%)	0.053	0.129	0.133	0.446
	2016–2017	16	16 (100%)	0.072	0.231	0.202	0.467
	2018–2019	31	31 (100%)	0.138	0.264	0.306	0.791
	2020–2021	32	32 (100%)	0.153	0.373	0.353	0.952
	2022–2023	16	16 (100%)	0.173	0.463	0.487	1.26
HMS-CA5	2012–2013	16	8 (50%)	<LOQ [0.02]	<0.020	<0.026	0.070
	2014–2015	16	10 (63%)	<LOQ [0.02]	0.022	<0.026	0.081
	2016–2017	16	11 (69%)	<LOQ [0.02]	0.028	<0.031	0.076
	2018–2019	31	27 (87%)	<LOQ [0.02]	0.052	<0.053	0.184
	2020–2021	32	29 (91%)	<LOQ [0.02]	0.055	<0.054	0.143
	2022–2023	16	16 (100%)	0.023	0.081	0.075	0.181

S5. Additional information on the statistic model

Male adults aged 61 years and older from the sample collection period 2012–2013 were used as reference as it was hypothesized that this group has the lowest usage of cosmetic products, including sunscreens. This hypothesis was in part based on the results of the recent 'sun protection behaviours survey' by the Australian Bureau of Statistic [11], which, inter alia, enquired about sunscreen usage on most days of the previous month.

Table S10. Initial regression model to identify potential predictors of biomarker concentrations in Australian pooled urine samples, determined using Tobit regression. The reference group is male, ≥61 years old, sample collection period 2012−2013. The interaction term was shown to add no significant improvement of the model for tHMS-CA and HMS-CA5 and was therefore excluded in the final model shown in Table 2 of the main manuscript for these two analytes.

	CPAA			5cx-EPS			tHMS-CA			HMS-CA5		
Factor	exp(β)	95% CI	p value	exp(β)	95% CI	p value	exp(β)	95% CI	p value	exp(β)	95% CI	p value
Intercept	4.69	(3.74–5.87)		0.171	(0.120–0.244)		0.0647	(0.0482–0.0868)		0.0076	(0.00517–0.0112)	
Sex=Male	1	Ref.	Ref.	1	Ref.	Ref.	1	Ref.	Ref.	1	Ref.	Ref.
Sex=Female	2.05	(1.59–2.64)	**<0.001**	3.69	(2.48–5.49)	**<0.001**	1.21	(0.874–1.69)	0.248	1.80	(1.21–2.69)	**0.004**
Age												
≥0–<5	19.6	(15.0–25.4)	**<0.001**	22.3	(14.7–33.8)	**<0.001**	5.50	(3.91–7.74)	**<0.001**	32.4	(21.6–48.6)	**<0.001**
≥5–<15	12.3	(9.58–15.9)	**<0.001**	7.75	(5.20–11.5)	**<0.001**	2.88	(2.07–3.99)	**<0.001**	13.0	(8.74–19.2)	**<0.001**
≥15–<30	2.15	(1.67–2.77)	**<0.001**	1.82	(1.22–2.72)	0.003	1.02	(0.733–1.42)	0.912	1.92	(1.28–2.86)	**0.001**
≥30–<45	2.61	(2.03–3.36)	**<0.001**	2.08	(1.40–3.10)	**<0.001**	1.63	(1.17–2.26)	**0.004**	2.67	(1.80–3.95)	**<0.001**
≥45–<60	2.37	(1.84–3.05)	**<0.001**	2.22	(1.49–3.31)	**<0.001**	1.50	(1.08–2.09)	**0.015**	2.26	(1.51–3.37)	**<0.001**
≥61	1	Ref.	Ref.	1	Ref.	Ref.	1	Ref.	Ref.	1	Ref.	Ref.
*Age*Sex*												
F; ≥0–<5	0.539	(0.372–0.780)	**0.001**	0.252	(0.141–0.452)	**<0.001**	0.820	(0.507–1.33)	0.417	0.562	(0.324–0.976)	**0.041**
F; ≥5–<15	0.470	(0.329–0.672)	**<0.001**	0.295	(0.168–0.519)	**<0.001**	0.872	(0.548–1.39)	0.562	0.635	(0.372–1.08)	0.096
F; ≥15–<30	0.546	(0.381–0.783)	**<0.001**	0.460	(0.260–0.811)	**0.007**	1.09	(0.680–1.74)	0.728	0.837	(0.485–1.44)	0.522
F; ≥30–<45	0.693	(0.485–0.990)	**0.044**	0.777	(0.442–1.36)	0.379	1.12	(0.702–1.78)	0.642	0.840	(0.492–1.44)	0.525
F; ≥45–<60	0.590	(0.413–0.843)	**0.004**	0.464	(0.264–0.816)	**0.008**	0.902	(0.567–1.43)	0.663	0.607	(0.353–1.04)	0.071
Year												
2012–2013	1	Ref.	Ref.	1	Ref.	Ref.	1	Ref.	Ref.	1	Ref.	Ref.
2014–2015	1.14	(0.924–1.40)	0.226	1.16	(0.84–1.61)	0.367	1.47	(1.12–1.92)	**0.005**	1.30	(0.951–1.78)	0.100
2016–2017	1.83	(1.47–2.27)	**<0.001**	1.50	(1.06–2.12)	**0.021**	2.23	(1.68–2.96)	**<0.001**	1.59	(1.14–2.22)	**0.006**
2018–2019	2.87	(2.40–3.43)	**<0.001**	2.03	(1.53–2.69)	**<0.001**	3.56	(2.82–4.49)	**<0.001**	3.19	(2.43–4.19)	**<0.001**
2020–2021	2.97	(2.48–3.55)	**<0.001**	1.78	(1.34–2.36)	**<0.001**	4.00	(3.17–5.04)	**<0.001**	3.22	(2.46–4.22)	**<0.001**
2022–2023	4.06	(3.30–4.99)	**<0.001**	1.96	(1.39–2.76)	**<0.001**	5.32	(4.07–6.96)	**<0.001**	4.33	(3.19–5.88)	**<0.001**

Table S11. The Likelihood-ratio-test showed that the age*sex interaction term significantly improved the model only for CPAA and 5cx-EPS, but not for tHMS-CA and HMS-CA5

Biomarker	$LL_{restricted}$	LL_{full}	$LR = -2 \cdot (LL_{restricted} - LL_{full})$	p value
CPAA	−86.762	−76.716	20.092	0.001
5cx-EPS	−177.138	−161.894	30.488	<0.001
tHMS-CA	−127.400	−126.082	2.637	0.76
HMS-CA5	−142.130	−138.731	6.798	0.24

LL: Log Likelihood, LR: Likelihood Ratio

Table S12. Alternative regression models for 5cx-EPS treating year as continuous variable in the forms of $x^{1/2}$, ln(x), and x^{-1} (Δ_i = 1.0, 0.0, and 1.5, respectively).

	Year modelled in the form of $x^{1/2}$				Year modelled in the form of ln(x)				Year modelled in the form of x^{-1}			
Factor	exp(β)	95% CI	p value		exp(β)	95% CI	p value		exp(β)	95% CI	p value	
Intercept	0.106	(0.0675–0.166)			0.167	(0.119–0.233)			0.375	(0.276–0.511)		
Age												
≥0–<5	22.3	(14.6–33.9)	**<0.001**		22.4	(14.7–34.0)	**<0.001**		22.6	(14.9–34.4)	**<0.001**	
≥5–<15	7.75	(5.17–11.6)	**<0.001**		7.75	(5.17–11.6)	**<0.001**		7.75	(5.16–11.6)	**<0.001**	
≥15–<30	1.82	(1.22–2.74)	**0.004**		1.82	(1.22–2.73)	**0.004**		1.82	(1.22–2.74)	**0.004**	
≥30–<45	2.08	(1.39–3.12)	**<0.001**		2.08	(1.39–3.12)	**<0.001**		2.08	(1.39–3.12)	**<0.001**	
≥45–<60	2.22	(1.48–3.33)	**<0.001**		2.22	(1.48–3.33)	**<0.001**		2.22	(1.48–3.33)	**<0.001**	
≥61	1	Ref.	Ref.		1	Ref.	Ref.		1	Ref.	Ref.	
Sex=Male	1	Ref.	Ref.		1	Ref.	Ref.		1	Ref.	Ref.	
Sex=Female	3.69	(2.46–5.53)	**<0.001**		3.69	(2.46–5.52)	**<0.001**		3.69	(2.46–5.53)	**<0.001**	
*Age*Sex*				*Age-specific sex effects*[a]				*Age-specific sex effects*[a]				*Age-specific sex effects*[a]
F; ≥0–<5	0.252	(0.139–0.475)	**<0.001**	0.930	0.252	(0.140–0.456)	**<0.001**	0.930	0.252	(0.139–0.475)	**<0.001**	0.930
F; ≥5–<15	0.295	(0.167–0.524)	**<0.001**	1.09	0.295	(0.167–0.523)	**<0.001**	1.09	0.295	(0.166–0.525)	**<0.001**	1.09
F; ≥15–<30	0.454	(0.255–0.810)	**0.007**	1.68	0.455	(0.256–0.810)	**0.007**	1.68	0.455	(0.255–0.813)	**0.008**	1.68
F; ≥30–<45	0.777	(0.438–1.38)	0.387	2.87	0.777	(0.438–1.38)	0.386	2.87	0.777	(0.437–1.38)	0.388	2.87
F; ≥45–<60	0.464	(0.262–0.824)	**0.009**	1.71	0.464	(0.262–0.822)	**0.009**	1.71	0.464	(0.262–0.824)	**0.009**	1.71
F; ≥61	1	Ref.	Ref.	3.69	1	Ref.	Ref.	3.69	1	Ref.	Ref.	3.69
$Year^{1/2}$ / ln(year) / $year^{-1}$	1.66	(1.38-1.99)	**<0.001**		1.52	(1.31-1.76)	**<0.001**		0.421	(0.306-0.579)	**<0.001**	

[a] $exp(\beta_{Sex}) \times exp(\beta_{Age*Sex})$

Table S13. Alternative regression models for tHMS-CA treating year as continuous variable in the forms of $x^{1/2}$ and ln(x) (Δ_i = 0.0 and 3.2, respectively).

	Year modelled in the form of $x^{1/2}$			Year modelled in the form of ln(x)		
Factor	exp(β)	95% CI	p value	exp(β)	95% CI	p value
Intercept	0.0197	(0.0141–0.0277)		0.0585	(0.0462–0.0741)	
Age						
≥0–<5	5.01	(3.92–6.40)	**<0.001**	5.07	(3.96–6.49)	**<0.001**
≥5–<15	2.69	(2.12–3.40)	**<0.001**	2.69	(2.12–3.41)	**<0.001**
≥15–<30	1.06	(0.832–1.34)	0.652	1.06	(0.832–1.35)	0.644
≥30–<45	1.72	(1.36–2.18)	**<0.001**	1.72	(1.36–2.18)	**<0.001**
≥45–<60	1.43	(1.13–1.81)	**0.003**	1.43	(1.13–1.81)	**0.003**
≥61	1	Ref.	Ref.	1	Ref.	Ref.
Sex=Male	1	Ref.	Ref.	1	Ref.	Ref.
Sex=Female	1.17	(1.02–1.34)	**0.029**	1.17	(1.02–1.34)	**0.030**
$Year^{1/2}$ / ln(year)	3.26	(2.80-3.80)	**<0.001**	2.59	(2.29-2.94)	**<0.001**

S6. Implications for risk management – HMS-CA5 in comparison to tHMS-CA

HMS-CA5 concentrations exceeded the HBM-I and/or -II value in the majority of children's pooled urine samples. On the other hand, concentrations of the *trans*-HMS biomarker tHMS-CA at maximum corresponded to 65% of the respective HBM-I value. This is related to (i) differences in relative biological availabilities of *cis*- and *trans*-HMS for the dermal and oral pathways and (ii) the lack of toxicological data for *cis*- and *trans*-HMS in isolation. This shall be explained in detail:
(i) For the oral uptake route, *cis*-HMS is a factor of 10 less biologically available than *trans*-HMS, whereas this difference is only a factor of 2 for the dermal route. Metabolism on the other hand is unaffected by the uptake route, which is why HBM data accurately reflects the systemic exposure to *cis*- and *trans*-HMS, irrespective of the uptake route (discussed in detail in Ebert et al. (2024) [3]).
(ii) All published toxicity studies on HMS have been performed with mixtures of the *cis*- and *trans*-diastereomers; therefore, their individual contributions to HMS toxicity are unknown – a shortcoming from which all risk assessments for HMS suffer (and not only HBM approaches). Accordingly, the point of departure for the derivation of the HBM-I and -II values for the HMS biomarkers was also based on an oral dosing study with a mixture of *cis*- and *trans*-HMS in rats. Using human toxicokinetic data for the oral pathway and assuming the typical diastereomer mixture in HMS (90% *cis*-HMS, 10% *trans*-HMS [4]), this point of departure was translated to urinary concentrations of HMS-CA5 and tHMS-CA by the German HBM commission. Due to the differences in relative biological availabilities of *cis*- and *trans*-HMS for the oral and dermal pathways, HMS-CA5 concentrations at the HBM-I value after dermal uptake of a 90% *cis*-, 10% *trans*-HMS mixture are expected to be accompanied by tHMS-CA concentrations being a factor of 5 (i.e., the difference in relative biological availabilities between the oral and dermal uptake routes) lower than the HBM-I value.

S7. References

(1) Bury, D.; Modick-Biermann, H.; Leibold, E.; Brüning, T.; Koch, H. M. Urinary metabolites of the UV filter octocrylene in humans as biomarkers of exposure. *Arch. Toxicol.* **2019**, *93* (5), 1227–1238. DOI: 10.1007/s00204-019-02408-7. Published Online: Feb. 9, 2019.
(2) Bury, D.; Griem, P.; Wildemann, T.; Brüning, T.; Koch, H. M. Urinary metabolites of the UV filter 2-Ethylhexyl salicylate as biomarkers of exposure in humans. *Toxicology letters* **2019**, *309*, 35–41. DOI: 10.1016/j.toxlet.2019.04.001. Published Online: Apr. 3, 2019.
(3) Ebert, K. E.; Griem, P.; Weiss, T.; Brüning, T.; Hayen, H.; Koch, H. M.; Bury, D. Toxicokinetics of homosalate in humans after dermal application: applicability of oral-route data for exposure assessment by human biomonitoring. *Arch. Toxicol.* **2024**, *98* (5), 1383–1398. DOI: 10.1007/s00204-024-03704-7. Published Online: Mar. 14, 2024.
(4) Ebert, K. E.; Griem, P.; Weiss, T.; Brüning, T.; Hayen, H.; Koch, H. M.; Bury, D. Diastereoselective metabolism of homomenthyl salicylate (homosalate): Identification of relevant human exposure biomarkers. *Environ. Int.* **2022**, *170*, 107637. DOI: 10.1016/j.envint.2022.107637. Published Online: Nov. 17, 2022.
(5) Bury, D.; Brüning, T.; Koch, H. M. Determination of metabolites of the UV filter 2-ethylhexyl salicylate in human urine by online-SPE-LC-MS/MS. *J. Chromatogr. B: Analyt. Technol. Biomed. Life Sci.* **2019**, *1110-1111*, 59–66. DOI: 10.1016/j.jchromb.2019.02.014.
(6) Bury, D.; Belov, V. N.; Qi, Y.; Hayen, H.; Volmer, D. A.; Brüning, T.; Koch, H. M. Determination of Urinary Metabolites of the Emerging UV Filter Octocrylene by Online-SPE-LC-MS/MS. *Anal. Chem.* **2018**, *90* (1), 944–951. DOI: 10.1021/acs.analchem.7b03996.
(7) Ebert, K. E.; Belov, V. N.; John, M.; Weiss, T.; Brüning, T.; Hayen, H.; Koch, H. M.; Bury, D. Identification, Organic Synthesis, and Sensitive Analysis of a cis-Homosalate-Specific Exposure Biomarker. *Chem. Res. Toxicol.* **2024**, *37* (2), 285–291. DOI: 10.1021/acs.chemrestox.3c00287. Published Online: Jan. 16, 2024.
(8) Ebert, K. E.; Belov, V. N.; Weiss, T.; Brüning, T.; Hayen, H.; Koch, H. M.; Bury, D. Determination of urinary metabolites of the UV filter homosalate by online-SPE-LC-MS/MS. *Anal. Chim. Acta* **2021**, *1176*, 338754. DOI: 10.1016/j.aca.2021.338754. Published Online: Jun. 14, 2021.
(9) Bury, D.; Weber, T.; Ebert, K. E.; Zülz, S.; Brüning, T.; Koch, H. M.; Kolossa-Gehring, M. Increasing exposure to the UV filters octocrylene and 2-ethylhexyl salicylate in Germany from 1996 to 2020: Human biomonitoring in 24-h urine samples of the German Environmental Specimen Bank (ESB). *Environment international* **2023**, *182*, 108334. DOI: 10.1016/j.envint.2023.108334.
(10) *COMMISSION DECISION of 14 August 2002 implementing Council Directive 96/23/EC concerning the performance of analytical methods and the interpretation of results*:*2002/657/EC*, 2002.
(11) Australian Bureau of Statistics. *Sun protection behaviours*. https://www.abs.gov.au/statistics/health/health-conditions-and-risks/sun-protection-behaviours/latest-release (accessed 06/2026).
